# Stable approach based diagonal recurrent quantum neural networks for identification of nonlinear systems

**DOI:** 10.1038/s41598-026-37973-2

**Published:** 2026-03-05

**Authors:** Hossam Khalil, Osama Elshazly, Omar Shaheen

**Affiliations:** 1 Mechatronics Engineering Department, College of Engineering, October 6 University, Giza, Egypt; 2 Electronics and Communications Engineering Department, Mansoura Higher Institute of Engineering and Technology, Mansoura, Egypt; 3 Department of Industrial Electronics and Control Engineering, Faculty of Electronic Engineering, Menoufia University, Menouf, Egypt

**Keywords:** Dynamic system identification, Quantum neural networks, Diagonal recurrent quantum neural networks, Lyapunov stability analysis, Engineering, Mathematics and computing, Physics

## Abstract

Identification of nonlinear dynamics from input-output data is crucial in many fields where conventional linear models fail to capture nonlinear dynamics of complex systems. Although recurrent neural network architectures have the potential to deal with these problems, they often face limitations in stability, memory capacity, and convergence efficiency. Recent developments in quantum neural networks (QNNs) offer a promising alternative due to their inherent parallelism and high-dimensional processing power. However, the application of QNNs in dynamic nonlinear modeling is still underexplored, especially with regard to stability-guaranteed learning strategies. To address this gap, a novel Diagonal Recurrent Quantum Neural architecture with Lyapunov Stability (DRQNN-LS) has been developed, which combines the structural simplicity of diagonal recurrent networks harnessing the capabilities of quantum learning algorithms and the mathematical rigor of Lyapunov stability theory. Stable convergence and efficient parameter tuning are ensured by deriving adaptive learning rates through Lyapunov analysis. The proposed model is evaluated through three scenarios: a mathematical nonlinear system, a chaotic Henon map, and a practical DC motor system. Comparative analysis with other models demonstrates the exceptional capabilities of DRQNN-LS in terms of the RMSE, MSE, and FIT metrics. The obtained responses validate the effectiveness and robustness of DRQNN-LS for modeling highly nonlinear and real-world systems.

## Introduction

Identification of nonlinear systems faces some challenges because they have intricate nonlinear relationships when representing the physical model. The identification of such systems is necessary when there is not enough information available about the system being modeled. Since linear models are insufficient to accurately describe the behavior of complex systems, there is an urgent need to identify nonlinear systems in many practical situations. This vital field of system identification has been found across several fields inclusive of but not restricted to medical and chemical applications^1,2^.

Artificial neural networks (ANNs) received significant interest within the application to dynamic system identification and control, as they are capable of nonlinear mapping, learning, and adaptation. Hence, they have been adopted for complex systems that are difficult to be modeled using conventional methods^3^. Feed-forward neural networks (FFNNs) and recurrent neural networks (RNNs) are core fundamental architectures widely employed as heuristic computational techniques in order to identify a system’s nonlinearities, each with its own unique characteristics and applications. FFNNs are multilayer NNs; within them, data flows in a unidirectional manner from input to output layers without any loops or feedback connections. FFNNs are introduced in various static models, including radial basis function networks (RBFNs)^4^, multi-layered perceptrons (MLPs)^5^, etc. FFNNs are extensively employed in tasks including classification^6^, functional approximation^7^, pattern recognition^8^, identification^9^, and control^10^. Within the scope of nonlinear identification of dynamic processes, usually the current system response is influenced by previous inputs and outputs, which makes it difficult to be identified by using FFNNs because they do not have memory features in their architecture. This makes them suitable for identifying only static and linear systems efficiently. However, FFNNS are utilized in many applications in order to identify and control nonlinear processes with the help of trapped delay lines (TDL)^11^. Just knowing the systems’ order, the previous inputs and outputs are fed to FFNNs through the use of TDLs. This converts the static FFNNs to dynamic FFNNs^12^.

On the other hand, another structure of neural networks is the recurrent neural networks (RNNs), which are specifically developed to handle sequential information^13^, where the order of inputs matters, and which don’t suffer from those drawbacks due to their simple structure. Unlike FFNNs, RNNs have recurrent connections that allow them to store and process data regarding previous inputs and utilize it to influence the current output. This ability to maintain internal memory, or “hidden state,” makes RNNs particularly useful for tasks such as speech recognition^14^, system identification and control^15^, and time series analysis^16^. RNN can be a fully connected recurrent neural network (FCRNN) or a partially connected recurrent neural network (PCRNN)^17^. In the FCRNN structure, all feedback weights between different layers are taken into consideration, including self-feedback, but in PCRNN, there is only self-feedback for each node. One of PCRNN’s structures is the diagonal recurrent neural network (DRNN)^18^, which contains only a single hidden layer with self-feedback for each hidden neuron. This makes DRNN simpler even than PCRNN because it has a smaller number of weights, which means a smaller training time. Unlike traditional recurrent neural networks, where information flows in a sequential manner along the recurrent connections, DRNNs incorporate diagonal connections that allow for more parallelism in information processing. The diagonal connections in DRNNs enable more efficient and effective handling of long-term dependencies in sequential data. By allowing information to be transmitted diagonally across time steps, DRNNs can capture complex patterns and relationships over longer sequences, which is especially useful in modelling and control of nonlinear processes^19–22^.

Quantum neural networks (QNNs) represent an innovative learning paradigm, which merges quantum mechanics with NNs, aiming to boost improvements in learning and processing efficiency^23,24^. QNNs serve as a class of neural networks that use quantum bits (qubits) to replace classical bits, allowing the network to process and store information in superposition states, which enables parallel processing of data. This quantum advantage significantly boosts the network’s computational capacity, making it capable of handling more complex and high-dimensional data with faster convergence rates compared to classical neural networks^25^. Hence, QNN has gained attention as a promising model addressing difficulties involved in capturing complex dynamics in nonlinear systems.

### Related works

Several studies have proved the effectiveness of QNNs in nonlinear process identification. The work in^26^ developed hybrid quantum-classical neural network architecture. This research demonstrates that combining quantum computing with classical neural networks makes it possible to achieve more efficient learning and faster convergence rates, particularly for high-dimensional data. Similarly, Kak in^27^ proposed a quantum neural computing framework that could be applied to system identification, highlighting the advantages of quantum data analyzed in this context. Analysis within^28^ employed quantum-inspired NN using a memetic algorithm to enhance chaotic time series forecasting and approximation. This method combines the flexibility of quantum-inspired models with the evolutionary efficiency of memetic algorithms to achieve accurate predictions and robust approximations in complex and dynamic systems. A new learning method was introduced in^29^ based on Lyapunov stability with dynamic rates for learning in order to identify nonlinear processes. This integration of quantum computing with neural network techniques represents a promising advancement in the field, offering the potential for significant improvements in both the stability and adaptability of models used for nonlinear system identification. The work in^30^ explores a hybrid technique that combines fuzzy logic, wavelet transforms, and QNNs to address the challenge of power system stability. The inclusion of a quantum neural network adds another layer of computational power, taking advantage of quantum-inspired mechanisms like superposition and coherence to improve the network’s learning capabilities and its ability to model complex dynamics. The paper^31^ introduced the design of a QNN-based intelligent control paradigm for continuous stirred tank reactors (CSTR) that employs a modified particle swarm optimization paradigm. This approach enhances the control accuracy and efficiency by leveraging quantum neural networks for improved dynamic response and optimization in complex chemical processes. An advanced control strategy aimed at enhancing the precision and stability of induction motor position control was proposed in^32^. To overcome these obstacles, the authors propose a fast terminal sliding mode control (FTSMC) approach, which is known for its robustness and ability to drive the system states to the desired equilibrium in a limited duration. However, to overcome the chattering problem typically associated with sliding mode control and to adapt to the nonlinear dynamics of the motor, the FTSMC is integrated with an adaptive QNN (AQNN). In^33^, the authors proposed a model-free direct adaptive controller, which relies on a quantum-inspired fuzzy network. The adaptive controller is designed to learn and adjust its parameters online, using feedback from the system’s performance. This ensures that the control strategy remains effective even as the system’s dynamics evolve over time. By integrating QNNs with a multi-layer structure, the paper^34^ introduces a controller that harnesses the computational power of quantum principles. The controller is trained using a real-coded genetic algorithm (RCGA), a type of evolutionary algorithm well-suited for optimizing continuous variables. The use of a multi-layer structure allows the QNN to capture intricate relationships within the data, enhancing the controller’s decision-making capabilities. The paper^35^ explores a novel approach to controlling robotic manipulators by leveraging the principles of QNNs, which are employed to model and control the dynamics of robotic manipulators to overcome challenges of traditional control in dealing with the nonlinearities and high degrees of freedom inherent in these systems. QNN-based controllers can outperform traditional neural network controllers in terms of response time, accuracy, and robustness. The authors in^36^ proposed a quantum-inspired NN representation that utilized quantum superposition and coherence, enhancing the learning and adaptation features of the network. Their research showed that QNNs could potentially outperform classical control systems, particularly in applications where the system dynamics were complex and constantly changing. As in^37^, QNN’s multi-layer architecture provides a robust framework for capturing intricate nonlinear relationships within the plasma system, which is crucial for maintaining stable operation. To optimize the QNN controller’s effectiveness, enhanced particle swarm optimization (PSO) algorithms are employed. By integrating improved PSO with a multi-layer QNN, the controller can more accurately and adaptively manage the chaotic dynamics of the plasma torch system, ensuring better stability and performance in various operational conditions. Additionally, quantum Boltzmann machines (QBMs) represent another key area of research within the realm of QNNs. QBMs extend classical Boltzmann machines by incorporating quantum elements, which can lead to more powerful generative models. Amin^38^ demonstrated that QBMs could outperform classical Boltzmann machines in certain tasks, such as sampling from complex probability distributions. These advances suggest that QBMs could have a vital impact on the future of quantum-enhanced NNs, notably in the field of unsupervised learning and system identification. A quantum-interference artificial neural network (QIANN) introduced in^39^ enhances space manipulator control by utilizing quantum interference to improve learning efficiency and precision, enabling more accurate and responsive control in complex space environments. Even though the rapidly expanding field of QNNs has seen significant advancements, it is currently unclear if QNNs are more potent than traditional NNs. The authors in^40^ used QNN to enhance the efficacy of maximum power point tracking (MPPT) frameworks for wind turbine systems. They focused on two widely used MPPT methods: tip-speed ratio and optimum torque. By integrating QNNs, they enhanced the tracking precision and operational efficiency of these methods. The work in^41^, introduced a Lyapunov-based physics-informed LSTM for adaptive control, where known system dynamics and Lyapunov stability constraints are embedded directly into the learning process. This integration guarantees closed-loop stability while improving robustness and tracking accuracy compared to purely data-driven recurrent models. Their work demonstrates that Lyapunov-guided learning significantly enhances the reliability of recurrent neural network–based adaptive control.

### Motivation

The diagonal recurrent structure is adopted in this study as a deliberate design choice to balance modeling capability, stability, and computational efficiency. Unlike fully recurrent networks, which suffer from strong parameter coupling and quadratic growth in recurrent weights, the diagonal recurrent architecture significantly reduces computational complexity and facilitates analytical tractability. This property is particularly important for Lyapunov-based learning, where clear error dynamics and negative definiteness of the Lyapunov function derivative must be guaranteed. Moreover, diagonal recurrence preserves the essential temporal memory required for nonlinear dynamic system identification while avoiding unstable or chaotic internal dynamics that may arise in fully recurrent structures. As a result, the diagonal recurrent formulation enables stable, efficient, and real-time–feasible learning, making it well suited for the proposed DRQNN-LS framework.

The Lyapunov-derived online update laws for fully recurrent quantum neural network were first presented in^42^. In this manuscript, we extend that methodology by: (1) applying a diagonal recurrent quantum structure (DRQNN) with a simplified diagonal recurrent configuration in which each hidden neuron includes only a self-feedback (diagonal) connection instead of full recurrent interconnections employed in^42^, thereby reducing the number of trainable parameters and improving training stability; (2) providing full derivations for the mathematical proof showing how the Lyapunov function leads to the adaptive learning rule for the proposed DRQNN algorithm; (3) validating on three different benchmark systems including an experimental DC-motor.

In particular, the field of application of QNNs for modeling and controlling nonlinear systems is yet open. In this research work, diagonal recurrent quantum neural network (DRQNN) based on Lyapunov stability theory is introduced, which is an integrated model that merges the features of DRNN and QNN. A combination of two powerful concepts, the Diagonal Recurrent Quantum Neural Network (DRQNN) and Lyapunov stability theory, has shown promise in modeling nonlinear systems with improved stability and convergence properties. The DRQNN leverages the strength of quantum computing to capture the intricate dynamics of nonlinear processes, while Lyapunov stability theory provides a stable learning algorithm for ensuring the stability of the identification technique. The integration of Lyapunov stability theory into the training phase of DRQNNs offers a valuable framework for modeling nonlinear systems with enhanced stability and convergence properties. This study demonstrates the effectiveness of the combined approach in capturing complex dynamics, including mathematical systems, chaotic series prediction, and real-world data from DC motors. This offers valuable insights into the potential of DRQNNs utilizing Lyapunov stability theory for nonlinear process modeling.

### Novelties and contributions

The key contributions in this research include:

1. A novel DRQNN-LS identification technique is developed for nonlinear systems. The computational efficiency of the DRNN is enhanced with the quantum computation and therefore the flexibility of the modeling technique design has been increased for handling the nonlinear dynamics offering superior modeling flexibility compared to DRNN-LS.

2. Distinct advancement over QNNs based Lyapunov learning: The proposed technique embeds Lyapunov stability conditions directly into a recurrent quantum neural framework, enabling stable learning for the network parameters. Tuning the extra parameters related to quantum computation increases the flexibility of the modeling strategy to approximate highly nonlinear dynamics.

3. The convergence has been satisfied through updating the parameters using adaptive learning rates which developed based on Lyapunov stability criteria.

4. Comprehensive validation across benchmarks and real data: The effectiveness of the proposed DRQNN-LS is demonstrated through nonlinear system modeling, time-series prediction, and real-world DC motor data. The results are systematically compared against DRNN-GD, DRNN-LS, and DRQNN-GD and the effectiveness of DRQNN-LS model for nonlinear identification is evaluated.

The remainder of this paper is structured as follows: Sect. “Problem overview” outlines the architecture of the proposed DRQNN model. In Sect. "Structure of the proposed DRQNN model", the learning algorithm is presented along with a comprehensive description of the associated learning rules for the DRQNN model parameters. Section "Learning algorithm for DRQNN model" discusses the analysis of convergence and stability for the DRQNN identification structure. Section "Stability analysis of RQNN identification model" presents the modeling results and compares them with alternative modeling strategies. Finally, Sect. “Simulation results” concludes the paper, with the references listed thereafter.

## Problem overview

A nonlinear dynamic plant can be generally expressed through a difference equation of the form:

1 $$\:{\mathcal{y}}_{p}\left(\varkappa\:\right)=\mathcal{f}\left({\mathcal{y}}_{p}\left(\varkappa\:-1\right),\:{\mathcal{y}}_{p}\left(\varkappa\:-2\right),\:\cdots\:,{\mathcal{y}}_{p}\left(\varkappa\:-\varrho\:+1\right),\:\mathcal{u}\left(\varkappa\:\right),\:\mathcal{u}\left(\varkappa\:-1\right),\:\cdots\:,\mathcal{u}\left(\varkappa\:-\rho\:+1\right)\right)$$

In this representation, $\:{\mathcal{y}}_{p}\left(\varkappa\:\right)$ is the system’s current output, which is influenced by its own previous outputs and the current and previous values of the external input $\:\mathcal{u}\left(\varkappa\:\right)$. The parameters $\:\varrho\:$ and $\:\rho\:$ denote the number of past output and input values considered, respectively. The function $\:\mathcal{f}$, which characterizes the system’s behavior, is assumed to be differentiable but unknown.

The major task is to approximate this nonlinear function $\:\mathcal{f}$ using the proposed DRQNN model, ensuring that the model output closely tracks the actual system response. The model’s dynamic behavior is captured by the following equation:

2 $$\:{\stackrel{\sim}{\mathcal{y}}}_{N}\left(\varkappa\:\right)=\widehat{\mathcal{f}}\left({\mathcal{y}}_{p}\left(\varkappa\:-1\right),\:{\mathcal{y}}_{p}\left(\varkappa\:-2\right),\:\cdots\:,{\mathcal{y}}_{p}\left(\varkappa\:-\varrho\:+1\right),\:\mathcal{u}\left(\varkappa\:\right),\:\mathcal{u}\left(\varkappa\:-1\right),\:\cdots\:,\mathcal{u}\left(\varkappa\:-\rho\:+1\right)\right)$$

Here, $\:{\stackrel{\sim}{\mathcal{y}}}_{N}\left(\varkappa\:\right)$ is the predicted output obtained with the DRQNN model, and $\:\widehat{\mathcal{f}}$ here is the model’s approximation of the actual system function $\:\mathcal{f}$. Through training, this approximation is refined over time such that:

3 $$\:\underset{\varkappa\:\to\:{\infty\:}}{\mathrm{lim}}\left|{\mathcal{y}}_{p}\left(\varkappa\:\right)-{\stackrel{\sim}{\mathcal{y}}}_{N}\left(\varkappa\:\right)\right|\le\:\epsilon\:$$

This indicates that, as the learning progresses, the model output converges to the actual system output within a small predefined error bound $\:\epsilon\:$.

## Structure of the proposed DRQNN model

**Fig. 1:**
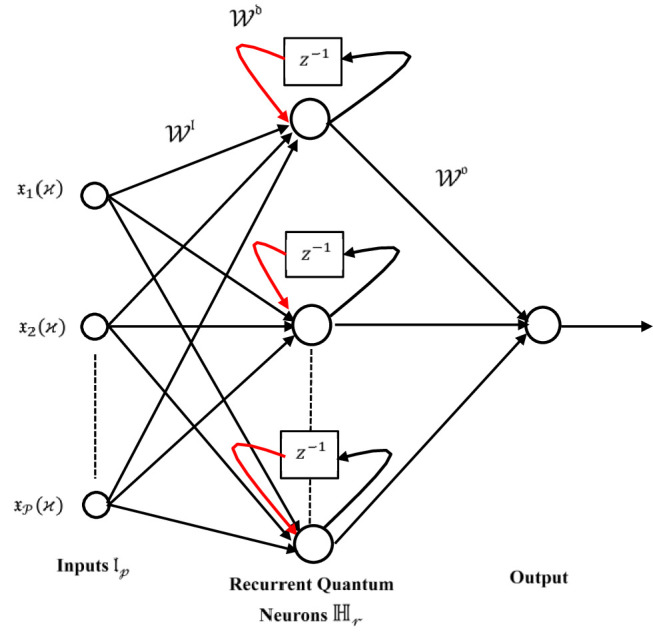
The developed DRQNN model architecture.

The DRQNN model integrates the features of recurrent neural networks and quantum computing to improve computational performance. By utilizing qubit-based neurons governed by quantum logic gate operations, the model effectively maps quantum states to neuron states. DRQNN is designed as a three-layer network with multiple inputs and a single output. The developed DRQNN is intended for modeling and identifying nonlinear system dynamics. Within Fig. 1, diagonal weights are depicted through a red arrow that transfers a delayed recurrent neuron output to be an input to the same recurrent neuron. $\:{\mathcal{W}}^{\mathfrak{d}}$ stands for the diagonal weight vector, $\:{\mathcal{W}}^{\mathrm{I}}$ stands for the input weight vector, and finally, $\:{\mathcal{W}}^{\mathfrak{o}}$ represents the output weight vector. All these weight vectors are adjusted based on Lyapunov theory.

The architecture of the developed DRQNN comprises three primary layers: input, hidden, and output, as illustrated in Fig. 1. The input layer ($\:{\mathfrak{l}}_{\mathcal{p}}$) accepts normalized inputs $\:{\mathfrak{x}}_{\mathcal{p}}\left(\varkappa\:\right)\in\:\left[0,\:1\right]$, which are transformed into quantum phase states using the relation:

4 $$\:{\mathcal{y}}_{\mathcal{p}}^{\mathfrak{l}}\left(\varkappa\:\right)=\frac{\pi\:}{2}{\mathfrak{x}}_{\mathcal{p}}\left(\varkappa\:\right)$$

Here $\:{\mathfrak{x}}_{\mathcal{p}}\left(\varkappa\:\right)$ denotes the $\:\mathcal{p}-th$ input signal, while$\:\:{\mathcal{y}}_{\mathcal{p}}^{\mathfrak{l}}\left(\varkappa\:\right)$ represents its quantum-phase equivalent. These phase values are then mapped to complex quantum states, and the output of the input layer is obtained as

5 $$\:{\mathcal{z}}_{\mathcal{p}}^{\mathfrak{l}}\left(\varkappa\:\right)=\mathcal{F}\left({\mathcal{y}}_{\mathcal{p}}^{\mathfrak{l}}\left(\varkappa\:\right)\right)={e}^{i\left({\mathcal{y}}_{\mathcal{p}}^{\mathfrak{l}}\left(\varkappa\:\right)\right)}=cos\left({\mathcal{y}}_{\mathcal{p}}^{\mathfrak{l}}\left(\varkappa\:\right)\right)+isin\left({\mathcal{y}}_{\mathcal{p}}^{\mathfrak{l}}\left(\varkappa\:\right)\right)$$

The imaginary and real components represent the probabilities of the qubit being in the firing $\:\left|1\rangle\right.$ and non-firing $\:\left|0\rangle\right.$ states, respectively. Moving to the hidden layer ($\:{\mathbb{H}}_{\mathcal{r}}$), it includes $\:\mathcal{R}$ recurrent quantum neurons. Each neuron incorporates feedback from its previous state through a diagonal weight $\:{\mathcal{W}}_{\mathcal{r}}^{\mathfrak{d}}$, enabling memory within the network. The neuron output is defined similarly to the input layer:

6 $$\:{\:\mathcal{z}}_{\mathcal{r}}^{\mathbb{H}}\left(\varkappa\:\right)=\mathcal{F}\left({\mathcal{y}}_{\mathcal{r}}^{\mathbb{H}}\left(\varkappa\:\right)\right)={e}^{i\left({\mathcal{y}}_{\mathcal{r}}^{\mathbb{H}}\left(\varkappa\:\right)\right)}=cos\left({\mathcal{y}}_{\mathcal{r}}^{\mathbb{H}}\left(\varkappa\:\right)\right)+isin\left({\mathcal{y}}_{\mathcal{r}}^{\mathbb{H}}\left(\varkappa\:\right)\right)$$

The internal phase $\:{\mathcal{y}}_{\mathcal{r}}^{\mathbb{H}}\left(\varkappa\:\right)$ is computed by combining the input contributions and recurrent feedback as

7 $$\:{\mathcal{y}}_{\mathcal{r}}^{\mathbb{H}}\left(\varkappa\:\right)=\frac{\pi\:}{2}\psi\:\left({\varrho\:}_{\mathcal{r}}\right)-arctan\left(\frac{{\sum\:}_{\mathcal{p}=1}^{\mathcal{P}}{\mathcal{W}}_{\mathcal{r}\mathcal{p}}^{\mathfrak{l}}\:sin\left({\mathcal{y}}_{\mathcal{p}}^{\mathfrak{l}}\left(\varkappa\:\right)\right)+{\mathcal{W}}_{\mathcal{r}}^{\mathfrak{d}}\:sin\left({\mathcal{y}}_{\mathcal{r}}^{\mathbb{H}}\left(\varkappa\:-1\right)\right)-sin\left({\phi\:}_{\mathcal{r}}\right)}{{\sum\:}_{\mathcal{p}=1}^{\mathcal{P}}{\mathcal{W}}_{\mathcal{r}\mathcal{p}}^{\mathfrak{l}}cos\left({\mathcal{y}}_{\mathcal{p}}^{\mathfrak{l}}\left(\varkappa\:\right)\right)+{\mathcal{W}}_{\mathcal{r}}^{\mathfrak{d}}\:cos\left({\mathcal{y}}_{\mathcal{r}}^{\mathbb{H}}\left(\varkappa\:-1\right)\right)-cos\left({\phi\:}_{\mathcal{r}}\right)}\right)$$

Here, $\:{\mathcal{W}}_{\mathcal{r}\mathcal{p}}^{\mathfrak{l}}$ are weights connecting the input to hidden neuron, $\:{\mathcal{W}}_{\mathcal{r}}^{\mathfrak{d}}$ is the recurrent (diagonal) weight, $\:{\phi\:}_{\mathcal{r}}\:$ is the phase bias, and $\:\psi\:\left({\varrho\:}_{\mathcal{r}}\right)$ is a sigmoid function within the range [0,1] that given as:

8 $$\:\psi\:\left({\varrho\:}_{\mathcal{r}}\right)\:=\frac{1}{1+{\boldsymbol{e}}^{-{\varrho\:}_{\mathcal{r}}}}$$

$\:{\varrho\:}_{\mathcal{r}}$ is the reversal factor equivalent to the NOT gate.

In the output layer ($\:\mathfrak{o}$), the combined hidden layer outputs determine the final output of DRQNN. The output state is then calculated as

9 $$\:{\mathcal{z}}^{\mathfrak{o}}=\mathbb{F}\left({\mathcal{y}}^{\mathfrak{o}}\right)={e}^{i{\mathcal{y}}^{\mathfrak{o}}}=cos\left({\mathcal{y}}^{\mathfrak{o}}\right)+isin\left({\mathcal{y}}^{\mathfrak{o}}\right)$$

The phase $\:{\mathcal{y}}^{\mathfrak{o}}$ is evaluated using:

10 $$\:{\mathcal{y}}^{\mathfrak{o}}=\frac{\pi\:}{2}\psi\:\left({\varrho\:}^{\mathfrak{o}}\right)-arctan\left(\frac{{\sum\:}_{\mathcal{r}=1}^{\mathcal{R}}{\mathcal{W}}_{\mathcal{r}}^{\mathfrak{o}}\:sin\left({\mathcal{y}}_{\mathcal{r}}^{\mathbb{H}}\left(\varkappa\:\right)\right)-sin\left({\phi\:}^{\mathfrak{o}}\right)}{{\sum\:}_{\mathcal{r}=1}^{\mathcal{R}}{\mathcal{W}}_{\mathcal{r}}^{\mathfrak{o}}\:\:cos\left({\mathcal{y}}_{\mathcal{r}}^{\mathbb{H}}\left(\varkappa\:\right)\right)-cos\left({\phi\:}^{\mathfrak{o}}\right)}\right)$$

where $\:{\mathcal{W}}_{\mathcal{r}}^{\mathfrak{o}}\:$ are the weights from the hidden to output layer, $\:{\phi\:}^{\mathfrak{o}}$ is the output node’s phase bias, and $\:{\varrho\:}^{\mathfrak{o}}$ serves as the reversal factor for the output neuron. The final output of the DRQNN model corresponds to the probability of the neuron being in the firing $\:\left|1\rangle\right.$ state, expressed as:

11 $$\:{\stackrel{\sim}{\mathcal{y}}}_{N}={\left|Im\left({\mathcal{z}}^{\mathfrak{o}}\right)\right|}^{2}$$

**Fig. 2:**
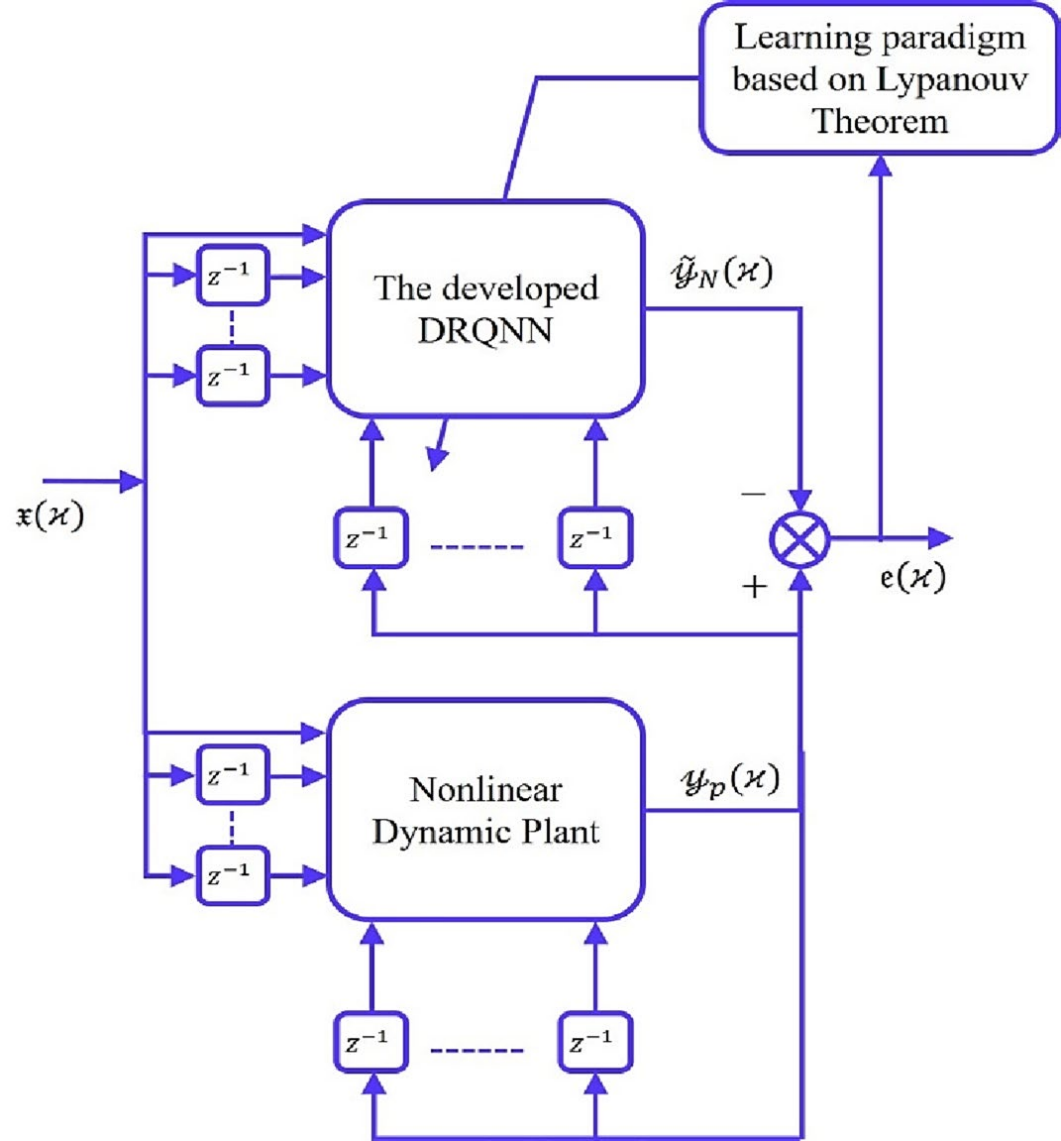
Identification Configuration DRQNN based model.

This structure is designed to efficiently approximate nonlinear system behavior, and its performance can be further enhanced by using an adaptive learning algorithm based on Lyapunov stability principles, which is detailed in the subsequent section. Figure 2 effectively represents the architecture of the process of nonlinear system identification using the DRQNN model. By employing quantum neuron states and recurrent feedback through delayed outputs, the DRQNN approximates the system dynamics. The error between the system output and DRQNN output is utilized to adapt the model parameters, ensuring stable learning through Lyapunov theory. This configuration leverages both quantum computing characteristics and neural memory mechanisms for efficient identification of complex nonlinear behaviors.

## Learning algorithm for DRQNN model

The main objective of the learning algorithm is to obtain the optimal values of DRQNN parameters, including the output layer parameters and hidden layer parameters. The learning theorem and Lyapunov-based derivations follow the approach presented in^42^. An efficient learning algorithm is developed, where the updating rules for the parameters of the proposed identification technique are derived based on Lyapunov stability theorem. Unlike conventional gradient-descent learning, which relies on heuristic step-size selection, the proposed Lyapunov-based rule provides a learning mechanism for weight adaptation with guaranteed stability, making the training process both reliable and interpretable for nonlinear dynamic system identification. The Lyapunov-based learning rule is derived by first defining the modeling error between the system output and the DRQNN output and then constructing a positive definite Lyapunov candidate function that includes both the error term and the parameter estimation errors. The time derivative of this Lyapunov function is analytically computed along the system trajectories, and the adaptive learning laws for the network weights are developed such that this derivative is negative semi-definite.

The developed Lyapunov-based learning is designed to minimize a predefined cost function, given by:

12 $$\:\stackrel{\sim}{\mathbb{E}}\left(\varkappa\:\right)=\frac{1}{2}{\left[\mathfrak{e}\left(\varkappa\:\right)\right]}^{2}={\left[{\mathcal{y}}_{p}\left(\varkappa\:\right)-{\stackrel{\sim}{\mathcal{y}}}_{N}\left(\varkappa\:\right)\right]}^{2}$$

The instantaneous modeling error $\:\mathfrak{e}\left(\varkappa\:\right)$ is calculated as the difference between the system output, $\:{\mathcal{y}}_{p}\left(\varkappa\:\right)$ and the DRQNN model output, $\:{\stackrel{\sim}{\mathcal{y}}}_{N}\left(\varkappa\:\right)\:$.

The learning algorithm derives the tuning relations for DRQNN parameters based on Lyapunov stability criteria. The parameter vector, which is updated, can be defined as $\:\mathcal{D}=\left[{\mathcal{W}}_{\mathcal{r}}^{\mathfrak{o}}\:{{\upphi\:}}^{\mathfrak{o}}\:{\varrho\:}^{\mathfrak{o}}\:\:{\mathcal{W}}_{\mathcal{r}\mathcal{p}}^{\mathfrak{l}}\:\:\:{\mathcal{W}}_{\mathcal{r}}^{\mathfrak{d}}\:\:\:{\varrho\:}_{\mathcal{r}}\:{{\upphi\:}}_{\mathcal{r}}\right]$, and the updating rule is defined as:

13 $$\:\mathcal{D}\left(\varkappa\:+1\right)=\mathcal{D}\left(\varkappa\:\right)-{\uplambda\:}\:{\Delta\:}\mathcal{D}\left(\varkappa\:\right)$$

Where $\:{\uplambda\:}\:$is the learning rate for parameter tuning. A Lyapunov stability theory is employed to conclude the general rule for updating the parameters of the proposed DRQNN model as provided in **Theorem** **1**.

### Theorem 1

With considering a positive definite Lyapunov function given by, $\:{\mathcal{V}}_{1}\left(\varkappa\:\right)={\upalpha\:}{\left(\mathfrak{e}\left(\varkappa\:\right)\right)}^{2}+{\upbeta\:}{\left(\:\mathcal{D}\left(\varkappa\:\right)\right)}^{2}>0$, where $\:{\upalpha\:}$ and $\:{\upbeta\:}$ are positive constants, the condition $\:{\mathcal{V}}_{1}\left(\varkappa\:\right)\le\:0$ is satisfied if and only if the adjustment term $\:{\Delta\:}\mathcal{D}\left(\varkappa\:\right)$ is obtained as:

14 $$\:{\Delta\:}\:\mathcal{D}\left(\varkappa\:\right)=-\frac{\mathcal{D}\left(\varkappa\:\right)+\frac{{\upalpha\:}}{{\upbeta\:}}\mathfrak{e}\left(\varkappa\:\right)\left(\frac{\partial\:\mathfrak{e}\left(\varkappa\:\right)}{\partial\:\mathcal{D}\left(\varkappa\:\right)}\right)}{1+\frac{{\upalpha\:}}{{\upbeta\:}}{\left(\frac{\partial\:\mathfrak{e}\left(\varkappa\:\right)}{\partial\:\mathcal{D}\left(\varkappa\:\right)}\right)}^{2}}$$

and consequently the parameters of the DRQNN model can be updated using the following general rule:

15 $$\:\mathcal{D}\left(\varkappa\:+1\right)=\:\mathcal{D}\left(\varkappa\:\right)-{\uplambda\:}\:{\Delta\:}\:\mathcal{D}\left(\varkappa\:\right)=\:\mathcal{D}\left(\varkappa\:\right)+{\uplambda\:}\:\frac{\:\mathcal{D}\left(\varkappa\:\right)+\frac{{\upalpha\:}}{{\upbeta\:}}\stackrel{\sim}{\mathrm{e}}\left(\varkappa\:\right)\left(\frac{\partial\:\mathfrak{e}\left(\varkappa\:\right)}{\partial\:\:\mathcal{D}\left(\varkappa\:\right)}\right)}{1+\frac{{\upalpha\:}}{{\upbeta\:}}{\left(\frac{\partial\:\mathfrak{e}\left(\varkappa\:\right)}{\partial\:\:\mathcal{D}\left(\varkappa\:\right)}\right)}^{2}}$$

### Proof 1

A Lyapunov function is initially selected as

16 $$\:{\mathcal{V}}_{1}\left(\varkappa\:\right)=\alpha\:{\left(\mathfrak{e}\left(\varkappa\:\right)\right)}^{2}+\beta\:{\left(\mathcal{D}\left(\varkappa\:\right)\right)}^{2}$$

The change of this function is obtained as

17 $$\:\varDelta\:{\mathcal{V}}_{1}\left(\varkappa\:\right)={\mathcal{V}}_{1}\left(\varkappa\:+1\right)-{\mathcal{V}}_{1}\left(\varkappa\:\right)=\left[\alpha\:{\left(\mathfrak{e}\left(\varkappa\:+1\right)\right)}^{2}+\beta\:{\left(\mathcal{D}\left(\varkappa\:+1\right)\right)}^{2}\right]-\:\left[\alpha\:{\left(\mathfrak{e}\left(\varkappa\:\right)\right)}^{2}+\beta\:{\left(\mathcal{D}\left(\varkappa\:\right)\right)}^{2}\right]$$

This equation can be rewritten as

18 $$\:\varDelta\:{\mathcal{V}}_{1}\left(\varkappa\:\right)=\alpha\:\left[\mathfrak{e}\left(\varkappa\:+1\right)+\mathfrak{e}\left(\varkappa\:\right)\right]\left[\mathfrak{e}\left(\varkappa\:+1\right)-\mathfrak{e}\left(\varkappa\:\right)\right]+\beta\:\left[\mathcal{D}\left(\varkappa\:+1\right)+\mathcal{D}\left(\varkappa\:\right)\right]\left[\mathcal{D}\left(\varkappa\:+1\right)-\mathcal{D}\left(\varkappa\:\right)\right]$$

Defining the differences

$$\:\varDelta\:\mathfrak{e}\left(\varkappa\:\right)=\mathfrak{e}\left(\varkappa\:+1\right)-\mathfrak{e}\left(\varkappa\:\right),\:\varDelta\:\mathcal{D}\left(\varkappa\:\right)=\mathcal{D}\left(\varkappa\:+1\right)-\mathcal{D}\left(\varkappa\:\right),$$.

Equation (17) can then be rewritten as

19 $$\:\varDelta\:{\mathcal{V}}_{1}\left(\varkappa\:\right)=\:\alpha\:\left[{\left(\varDelta\:\mathfrak{e}\left(\varkappa\:\right)\right)}^{2}+2\mathfrak{e}\left(\varkappa\:\right)\varDelta\:\mathfrak{e}\left(\varkappa\:\right)\right]+\beta\:\left[{\left(\varDelta\:\mathcal{D}\left(\varkappa\:\right)\right)}^{2}+2\mathcal{D}\left(\varkappa\:\right)\varDelta\:\mathcal{D}\left(\varkappa\:\right)\right]$$

20 $$\:\varDelta\:{\mathcal{V}}_{1}\left(\varkappa\:\right)={\left(\varDelta\:\mathcal{D}\left(\varkappa\:\right)\right)}^{2}\:\left[{\upbeta\:}+{\upalpha\:}{\left(\frac{\varDelta\:\mathfrak{e}\left(\varkappa\:\right)}{\varDelta\:\mathcal{D}\left(\varkappa\:\right)}\right)}^{2}\right]+2\varDelta\:\mathcal{D}\left(\varkappa\:\right)\left[{\upbeta\:}\mathcal{D}\left(\varkappa\:\right)+{\upalpha\:}\mathfrak{e}\left(\varkappa\:\right)\left(\frac{\varDelta\:\mathfrak{e}\left(\varkappa\:\right)}{\varDelta\:\mathcal{D}\left(\varkappa\:\right)}\right)\right]$$

With small changes, the above equation can be formulated as

21 $$\:\varDelta\:{\mathcal{V}}_{1}\left(\varkappa\:\right)={\left(\varDelta\:\mathcal{D}\left(\varkappa\:\right)\right)}^{2}\:\left[\beta\:+\alpha\:{\left(\frac{\partial\:\mathfrak{e}\left(\varkappa\:\right)}{\partial\:\mathcal{D}\left(\varkappa\:\right)}\right)}^{2}\right]+2\varDelta\:\mathcal{D}\left(\varkappa\:\right)\left[\beta\:\mathcal{D}\left(\varkappa\:\right)+\alpha\:\mathfrak{e}\left(\varkappa\:\right)\left(\frac{\partial\:\mathfrak{e}\left(\varkappa\:\right)}{\partial\:\mathcal{D}\left(\varkappa\:\right)}\right)\right]$$

Let

22 $$\:\varDelta\:{\mathcal{V}}_{1}\left(\varkappa\:\right)={\left(\varDelta\:\mathcal{D}\left(\varkappa\:\right)\right)}^{2}\:\left[\beta\:+\alpha\:{\left(\frac{\partial\:\mathfrak{e}\left(\varkappa\:\right)}{\partial\:\mathcal{D}\left(\varkappa\:\right)}\right)}^{2}\right]+2\varDelta\:\mathcal{D}\left(\varkappa\:\right)\left[\beta\:\mathcal{D}\left(\varkappa\:\right)+\alpha\:\mathfrak{e}\left(\varkappa\:\right)\left(\frac{\partial\:\mathfrak{e}\left(\varkappa\:\right)}{\partial\:\mathcal{D}\left(\varkappa\:\right)}\right)\right]=-\epsilon\:$$

where $\:\epsilon\:$ must have a positive or zero value in order for condition,$\:\:\varDelta\:{\mathcal{V}}_{1}\left(\varkappa\:\right)\le\:0$, to hold. So Eq. (22) becomes

23 $$\:{\left(\varDelta\:\mathcal{D}\left(\varkappa\:\right)\right)}^{2}\:\left[\beta\:+\alpha\:{\left(\frac{\partial\:\mathfrak{e}\left(\varkappa\:\right)}{\partial\:\mathcal{D}\left(\varkappa\:\right)}\right)}^{2}\right]+2\varDelta\:\mathcal{D}\left(\varkappa\:\right)\left[\beta\:\mathcal{D}\left(\varkappa\:\right)+\alpha\:\mathfrak{e}\left(\varkappa\:\right)\left(\frac{\partial\:\mathfrak{e}\left(\varkappa\:\right)}{\partial\:\mathcal{D}\left(\varkappa\:\right)}\right)\right]+\epsilon\:=0$$

Consider a general quadratic Eq. 

24 $$\:\mathcal{a}{\mathcal{x}}^{2}+\mathcal{b}\mathcal{x}+\mathcal{c}=0$$

The roots of the quadratic equation are given by

25 $$\:{\mathcal{r}}_{1}=\frac{-\mathcal{b}+\sqrt{{\mathcal{b}}^{2}-4\mathcal{a}\mathcal{c}}}{2\mathcal{a}}\:\mathrm{a}\mathrm{n}\mathrm{d}\:{\mathcal{r}}_{2}=\frac{-\mathcal{b}-\sqrt{{\mathcal{b}}^{2}-4\mathcal{a}\mathcal{c}}}{2\mathcal{a}}$$

Comparing Eq. (23) with Eq. (24), it can be easily seen that $\:\varDelta\:\mathcal{D}\left(\varkappa\:\right)$ acts as $\:\mathcal{x}$ in Eq. (24) and the values of $\:\mathcal{a},\:\mathcal{b}$ and $\:\mathcal{c}$ in Eq. (24) are defined as:

$$\:\mathcal{a}=\beta\:+\alpha\:{\left(\frac{\partial\:\mathfrak{e}\left(\varkappa\:\right)}{\partial\:\mathcal{D}\left(\varkappa\:\right)}\right)}^{2},\mathcal{b}=2\left[\beta\:\mathcal{D}\left(\varkappa\:\right)+\alpha\:\mathfrak{e}\left(\varkappa\:\right)\left(\frac{\partial\:\mathfrak{e}\left(\varkappa\:\right)}{\partial\:\mathcal{D}\left(\varkappa\:\right)}\right)\right],\:\mathrm{a}\mathrm{n}\mathrm{d}\:\mathcal{c}=\epsilon\:$$

To have a single unique solution of quadratic equation, term $\:\sqrt{{\mathcal{b}}^{2}-4\mathcal{a}\mathcal{c}}$ must be equal to zero. Putting values of $\:\mathcal{a},\:\mathcal{b}$ and $\:\mathcal{c}$ in $\:\sqrt{{\mathcal{b}}^{2}-4\mathcal{a}\mathcal{c}}$ we get

26 $$\:\sqrt{4{\left[\beta\:\mathcal{D}\left(\varkappa\:\right)+\alpha\:\mathfrak{e}\left(\varkappa\:\right)\left(\frac{\partial\:\mathfrak{e}\left(\varkappa\:\right)}{\partial\:\mathcal{D}\left(\varkappa\:\right)}\right)\right]}^{2}-4\left[\beta\:+\alpha\:{\left(\frac{\partial\:\mathfrak{e}\left(\varkappa\:\right)}{\partial\:\mathcal{D}\left(\varkappa\:\right)}\right)}^{2}\right]{\upepsilon\:}}=0$$

On squaring both sides we get

27 $$\:{\left[\beta\:\mathcal{D}\left(\varkappa\:\right)+\alpha\:\mathfrak{e}\left(\varkappa\:\right)\left(\frac{\partial\:\mathfrak{e}\left(\varkappa\:\right)}{\partial\:\mathcal{D}\left(\varkappa\:\right)}\right)\right]}^{2}-\left[\beta\:+\alpha\:{\left(\frac{\partial\:\mathfrak{e}\left(\varkappa\:\right)}{\partial\:\mathcal{D}\left(\varkappa\:\right)}\right)}^{2}\right]{\upepsilon\:}=0$$

So, $\:{\upepsilon\:}$ comes out to be

28 $$\:{\upepsilon\:}=\frac{{\left[\mathcal{D}\left(\varkappa\:\right)+\frac{\alpha\:}{\beta\:}\mathfrak{e}\left(\varkappa\:\right)\left(\frac{\partial\:\mathfrak{e}\left(\varkappa\:\right)}{\partial\:\mathcal{D}\left(\varkappa\:\right)}\right)\right]}^{2}}{\left[1+\frac{\alpha\:}{\beta\:}{\left(\frac{\partial\:\mathfrak{e}\left(\varkappa\:\right)}{\partial\:\mathcal{D}\left(\varkappa\:\right)}\right)}^{2}\right]}$$

Since $\:{\upepsilon\:}\ge\:0$ which means

29 $$\:\frac{{\left[\mathcal{D}\left(\varkappa\:\right)+\frac{\alpha\:}{\beta\:}\mathfrak{e}\left(\varkappa\:\right)\left(\frac{\partial\:\mathfrak{e}\left(\varkappa\:\right)}{\partial\:\mathcal{D}\left(\varkappa\:\right)}\right)\right]}^{2}}{\left[1+\frac{\alpha\:}{\beta\:}{\left(\frac{\partial\:\mathfrak{e}\left(\varkappa\:\right)}{\partial\:\mathcal{D}\left(\varkappa\:\right)}\right)}^{2}\right]}\ge\:0$$

So, the unique root of Eq. (23) will be given as

30 $$\:\varDelta\:\mathcal{D}\left(\varkappa\:\right)=\frac{-\mathcal{b}}{2\mathcal{a}}=-\frac{{\left[\mathcal{D}\left(\varkappa\:\right)+\frac{\alpha\:}{\beta\:}\mathfrak{e}\left(\varkappa\:\right)\left(\frac{\partial\:\mathfrak{e}\left(\varkappa\:\right)}{\partial\:\mathcal{D}\left(\varkappa\:\right)}\right)\right]}^{2}}{\left[1+\frac{\alpha\:}{\beta\:}{\left(\frac{\partial\:\mathfrak{e}\left(\varkappa\:\right)}{\partial\:\mathcal{D}\left(\varkappa\:\right)}\right)}^{2}\right]}$$

Hence, the updating equation that defined in Eq. (14) is obtained. This completes the proof.

This leads to the learning rule outlined in Eq. (15), which governs the adjustment of both hidden and output layer parameters within the developed DRQNN model.

**Fig. 3:**
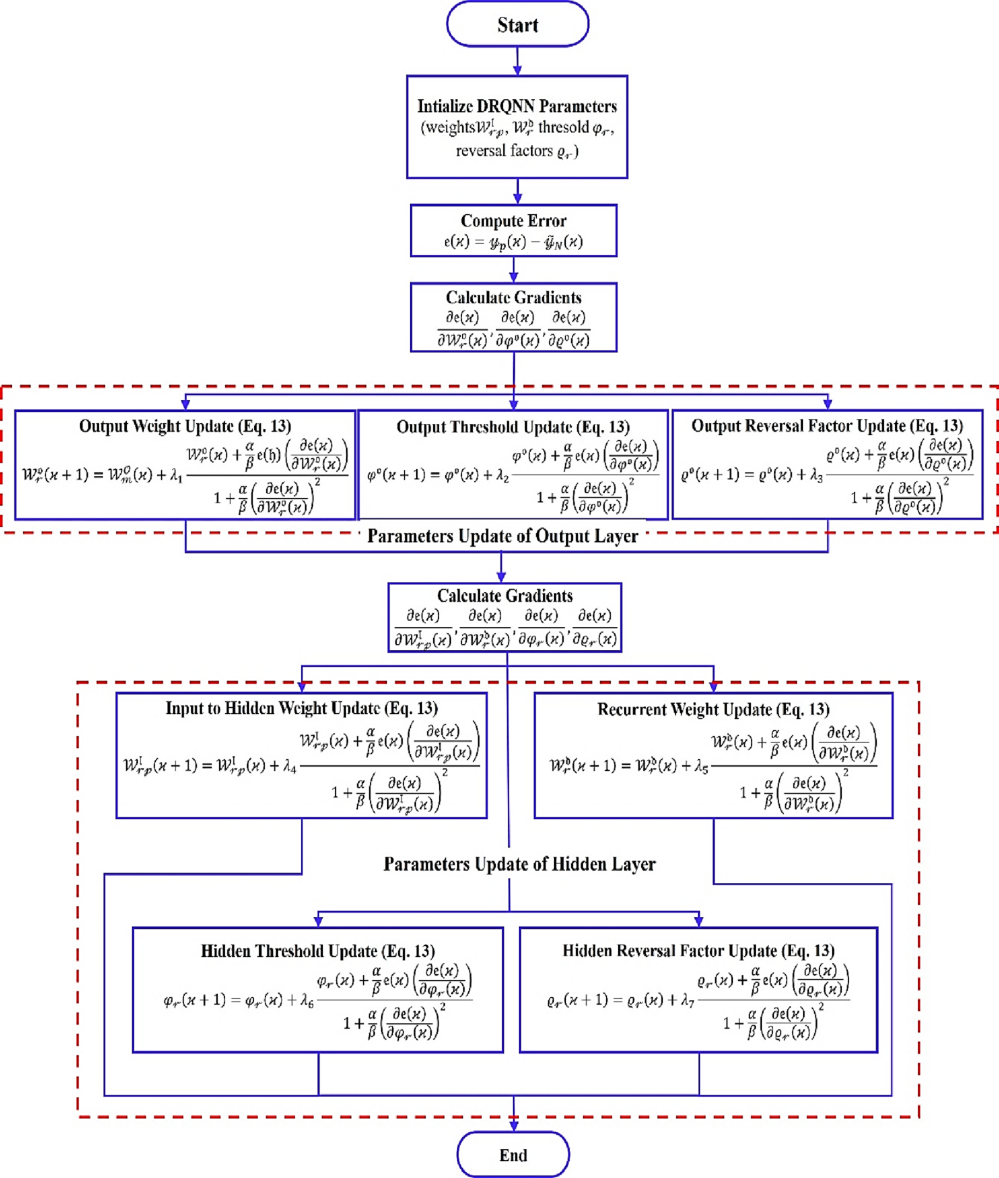
Learning algorithm for tuning the parameters of the DRQNN model.

The learning algorithm for tuning weights, threshold, and reversal parameters of the output and hidden layers is explained in the flowchart shown in Fig. 3. The starting point of the learning algorithm is calculating the error signal from the difference between the system output and the DRQNN model output. Then the gradients of the output layer parameters are calculated. The process then goes on to adjust the output layer parameters: weights, threshold, and reversal parameters. For tuning the parameters of the hidden layer, firstly the gradients of the hidden layer parameters are calculated, and adjustment processes of the weights connecting the input to the hidden neuron, recurrent weights, threshold, and reversal parameters are performed.

The learning rates $\:{\lambda\:}_{1},{\lambda\:}_{2}$ and $\:{\lambda\:}_{3}$ are used in the adjustment relations for output layer parameters. $\:{\lambda\:}_{4},{\lambda\:}_{5}$, $\:{\lambda\:}_{6}$, and $\:{\lambda\:}_{7}$ are the learning rates for updating the hidden layer parameters. The gradients of the output layer parameters are calculated as follows:

31 $$\:\frac{\partial\:\mathfrak{e}\left(\varkappa\:\right)}{\partial\:{\mathcal{W}}_{\mathcal{r}}^{\mathfrak{o}}\left(\varkappa\:\right)}=2\mathfrak{e}\left(\varkappa\:\right)\mathrm{sin}\left({\mathcal{y}}^{\mathfrak{o}}\left(\varkappa\:\right)\right)\mathrm{cos}\left({\mathcal{y}}^{\mathfrak{o}}\left(\varkappa\:\right)\right)\frac{{\mathcal{A}}^{\mathfrak{o}}\mathrm{sin}\left({\mathcal{y}}_{\mathcal{r}}^{\mathbb{H}}\left(\varkappa\:\right)\right)+{\mathcal{B}}^{\mathfrak{o}}\mathrm{cos}\left({\mathcal{y}}_{\mathcal{r}}^{\mathbb{H}}\left(\varkappa\:\right)\right)}{{\left({\mathcal{A}}^{\mathfrak{o}}\right)}^{2}+{\left({\mathcal{B}}^{\mathfrak{o}}\right)}^{2}}\:\:$$

32 $$\:\frac{\partial\:\mathfrak{e}\left(\varkappa\:\right)}{\partial\:{\phi\:}^{\mathfrak{o}}\left(\varkappa\:\right)}=-2\mathfrak{e}\left(\varkappa\:\right)\mathrm{s}\mathrm{i}\mathrm{n}\left({\mathcal{y}}^{\mathfrak{o}}\left(\varkappa\:\right)\right)\mathrm{c}\mathrm{o}\mathrm{s}\left({\mathcal{y}}^{\mathfrak{o}}\left(\varkappa\:\right)\right)\frac{{\mathcal{A}}^{\mathfrak{o}}\mathrm{sin}\left({\phi\:}^{\mathfrak{o}}\left(\varkappa\:\right)\right)+{\mathcal{B}}^{\mathfrak{o}}\mathrm{c}\mathrm{o}\mathrm{s}\left({\phi\:}^{\mathfrak{o}}\left(\varkappa\:\right)\right)}{{\left({\mathcal{A}}^{\mathfrak{o}}\right)}^{2}+{\left({\mathcal{B}}^{\mathfrak{o}}\right)}^{2}}\:\:$$

33 $$\:\frac{\partial\:\mathfrak{e}\left(\varkappa\:\right)}{\partial\:{\varrho\:}^{\mathfrak{o}}\left(\varkappa\:\right)}=-\pi\:\mathfrak{e}\left(\varkappa\:\right)\mathrm{s}\mathrm{i}\mathrm{n}\left({\mathcal{y}}^{\mathfrak{o}}\left(\varkappa\:\right)\right)\mathrm{c}\mathrm{o}\mathrm{s}\left({\mathcal{y}}^{\mathfrak{o}}\left(\varkappa\:\right)\right)\frac{{e}^{-{\varrho\:}^{\mathfrak{o}}}}{{\left(1+{e}^{-{\varrho\:}^{\mathfrak{o}}}\right)}^{2}}\:\:$$

where

34 $$\:{\:\mathcal{A}}^{\mathfrak{o}}={\sum\:}_{\mathcal{r}=1}^{\mathcal{R}}{\mathcal{W}}_{\mathcal{r}}^{\mathfrak{o}}\:\mathrm{s}\mathrm{i}\mathrm{n}({\mathcal{y}}_{\mathcal{r}}^{\mathbb{H}}\left(\varkappa\:\right))-\mathrm{s}\mathrm{i}\mathrm{n}({\phi\:}^{\mathfrak{o}})$$

35 $$\:{\mathcal{B}}^{\mathfrak{o}}={\sum\:}_{\mathcal{r}=1}^{\mathcal{R}}{\mathcal{W}}_{\mathcal{r}}^{\mathfrak{o}}\:\mathrm{c}\mathrm{o}\mathrm{s}({\mathcal{y}}_{\mathcal{r}}^{\mathbb{H}}\left(\varkappa\:\right))-\mathrm{c}\mathrm{o}\mathrm{s}({\phi\:}^{\mathfrak{o}})$$

Also, the gradients of the hidden layer parameters are calculated as follows:

36 $$\:\frac{\partial\:\mathfrak{e}\left(\varkappa\:\right)}{\partial\:{\mathcal{W}}_{\mathcal{r}\mathcal{p}}^{\mathfrak{l}}\left(\varkappa\:\right)}=-{\Phi\:}\left(\varkappa\:\right)\frac{{\mathcal{A}}_{\mathcal{r}}^{\mathbb{H}}\mathrm{sin}\left({\mathcal{y}}_{\mathcal{p}}^{\mathfrak{l}}\left(\varkappa\:\right)\right)-{\mathcal{B}}_{\mathcal{r}}^{\mathbb{H}}\mathrm{cos}\left({\mathcal{y}}_{\mathcal{p}}^{\mathfrak{l}}\left(\varkappa\:\right)\right)}{{\left({\mathcal{A}}_{\mathcal{r}}^{\mathbb{H}}\right)}^{2}+{\left({\mathcal{B}}_{\mathcal{r}}^{\mathbb{H}}\right)}^{2}}\:\:$$

37 $$\:\frac{\partial\:\mathfrak{e}\left(\varkappa\:\right)}{\partial\:{\mathcal{W}}_{\mathcal{r}}^{\mathfrak{d}}\left(\varkappa\:\right)}=-{\Phi\:}\left(\varkappa\:\right)\frac{{\mathcal{A}}_{\mathcal{r}}^{\mathbb{H}}\sum\:_{\mathcal{r}=1}^{\mathcal{R}}sin\left({\mathcal{y}}_{\mathcal{r}}^{\mathbb{H}}\left(\varkappa\:-1\right)\right)-{\mathcal{B}}_{\mathcal{r}}^{\mathbb{H}}\sum\:_{\mathcal{r}=1}^{\mathcal{R}}cos\left({\mathcal{y}}_{\mathcal{r}}^{\mathbb{H}}\left(\varkappa\:-1\right)\right)}{{\left({\mathcal{A}}_{\mathcal{r}}^{\mathbb{H}}\right)}^{2}+{\left({\mathcal{B}}_{\mathcal{r}}^{\mathbb{H}}\right)}^{2}}\:\:$$

38 $$\:\frac{\partial\:\mathfrak{e}\left(\varkappa\:\right)}{\partial\:{\phi\:}_{\mathcal{r}}\left(\varkappa\:\right)}=\varPhi\:\left(\varkappa\:\right)\frac{{\mathcal{A}}_{\mathcal{r}}^{\mathbb{H}}{cos}\left({\phi\:}_{\mathcal{r}}\right)+{\mathcal{B}}_{\mathcal{r}}^{\mathbb{H}}sin\left({\phi\:}_{\mathcal{r}}\right)}{{\left({\mathcal{A}}_{\mathcal{r}}^{\mathbb{H}}\right)}^{2}+{\left({\mathcal{B}}_{\mathcal{r}}^{\mathbb{H}}\right)}^{2}}\:\:$$

39 $$\:\frac{\partial\:\mathfrak{e}\left(\varkappa\:\right)}{\partial\:{\varrho\:}_{\mathcal{r}}\left(\varkappa\:\right)}=\frac{\pi\:}{2}{\Phi\:}\left(\varkappa\:\right)\frac{{e}^{-{\varrho\:}_{\mathcal{r}}}}{{\left(1+{e}^{-{\varrho\:}_{\mathcal{r}}}\right)}^{2}}\:\:$$

where

40 $$\:{\Phi\:}\left(\varkappa\:\right)=2\mathfrak{e}\left(\varkappa\:\right)\mathrm{s}\mathrm{i}\mathrm{n}\left({\mathcal{y}}^{\mathfrak{o}}\left(\varkappa\:\right)\right)\mathrm{c}\mathrm{o}\mathrm{s}\left({\mathcal{y}}^{\mathfrak{o}}\left(\varkappa\:\right)\right)\frac{{\mathcal{A}}^{\mathfrak{o}}{\mathcal{W}}_{\mathcal{r}}^{\mathfrak{o}}\mathrm{cos}\left({\mathcal{y}}_{\mathcal{r}}^{\mathbb{H}}\left(\varkappa\:\right)\right)+{\mathcal{B}}^{\mathfrak{o}}{\mathcal{W}}_{\mathcal{r}}^{\mathfrak{o}}\mathrm{s}\mathrm{i}\mathrm{n}\left({\mathcal{y}}_{\mathcal{r}}^{\mathbb{H}}\left(\varkappa\:\right)\right)}{{\left({\mathcal{A}}^{\mathfrak{o}}\right)}^{2}+{\left({\mathcal{B}}^{\mathfrak{o}}\right)}^{2}}$$

41 $$\:{\mathcal{A}}_{\mathcal{r}}^{\mathbb{H}}=\sum\:_{\mathcal{p}=1}^{\mathcal{P}}{\mathcal{W}}_{\mathcal{r}\mathcal{p}}^{\mathfrak{l}}\mathrm{sin}\left({\mathcal{y}}_{\mathcal{p}}^{\mathfrak{l}}\left(\varkappa\:\right)\right)+{\mathcal{W}}_{\mathcal{r}}^{\mathfrak{d}}sin\left({\mathcal{y}}^{\mathbb{H}}\left(\varkappa\:-1\right)\right)-\mathrm{s}\mathrm{i}\mathrm{n}({\phi\:}_{\mathcal{r}})\:$$

42 $$\:{\mathcal{B}}_{\mathcal{r}}^{\mathbb{H}}=\sum\:_{\mathcal{p}=1}^{\mathcal{P}}{\mathcal{W}}_{\mathcal{r}\mathcal{p}}^{\mathfrak{l}}\mathrm{cos}\left({\mathcal{y}}_{\mathcal{p}}^{\mathfrak{l}}\left(\varkappa\:\right)\right)+{\mathcal{W}}_{\mathcal{r}}^{\mathfrak{d}}cos\left({\mathcal{y}}^{\mathbb{H}}\left(\varkappa\:-1\right)\right)-\mathrm{c}\mathrm{o}\mathrm{s}({\phi\:}_{\mathcal{r}})$$

## Stability analysis of RQNN identification model

The stability and convergence speed are affected by the values of learning rates through the tuning of the parameters of the DRQNN model. Small values of such learning rates satisfy the convergence, but the training time will be long. Larger values ​​increase the learning speed, but this can lead to system instability. Therefore, learning rate values ​​must be carefully chosen to adjust the learning speed and thus ensure stability. Adaptive learning rates can be developed in **Theorem** **2** based on Lyapunov stability criteria^43^.

### Theorem 2

The stability can be guaranteed with adaptive learning rates if the following condition for the values of learning rates of the proposed DRQNN model is satisfied.

43 $$\:0\:\le\:\:{{\uplambda\:}}_{\mathcal{i}\:}\left(\varkappa\:\right)\le\:\frac{2}{{\left[\frac{\partial\:{\stackrel{\sim}{\mathcal{y}}}_{N}\left(\varkappa\:\right)}{\partial\:\mathcal{D}\left(\varkappa\:\right)}\right]}^{2}}$$

### Proof 2

Let a positive definite Lyapunov function be given by

44 $$\:{\mathcal{V}}_{2}\left(\varkappa\:\right)=\:\frac{1}{2}{\mathfrak{e}}^{2}\left(\varkappa\:\right)$$

The basic condition that ensures stability is

45 $$\:\varDelta\:{\mathcal{V}}_{2}\left(\varkappa\:\right)=\:{\mathcal{V}}_{2}\left(\varkappa\:+1\right)-{\mathcal{V}}_{2}\left(\varkappa\:\right)\le\:0$$

where $\:\varDelta\:{\mathcal{V}}_{2}\left(\varkappa\:\right)$ is the change in Lyapunov function and it is obtained as

46 $$\:\varDelta\:{\mathcal{V}}_{2}\left(\varkappa\:\right)=\frac{1}{2}\:\left({\mathfrak{e}}^{2}\left(\varkappa\:+1\right)-{\mathfrak{e}}^{2}\left(\varkappa\:\right)\right)$$

Or

47 $$\:\varDelta\:{\mathcal{V}}_{2}\left(\varkappa\:\right)=\frac{1}{2}\:\left[\mathfrak{e}\left(\varkappa\:+1\right)+\mathfrak{e}\left(\varkappa\:\right)\right]\left[\mathfrak{e}\left(\varkappa\:+1\right)-\mathfrak{e}\left(\varkappa\:\right)\right]$$

Let $\:\varDelta\:\mathfrak{e}\left(\varkappa\:\right)=\mathfrak{e}\left(\varkappa\:+1\right)-\mathfrak{e}\left(\varkappa\:\right)$, Eq. (47) can be written as

48 $$\:\varDelta\:{\mathcal{V}}_{2}\left(\varkappa\:\right)=\frac{1}{2}\:\left[\varDelta\:\mathfrak{e}\left(\varkappa\:\right)+2\mathfrak{e}\left(\varkappa\:\right)\right]\left[\varDelta\:\mathfrak{e}\left(\varkappa\:\right)\right]=\varDelta\:\mathfrak{e}\left(\varkappa\:\right)\left[\frac{1}{2}\:\varDelta\:\mathfrak{e}\left(\varkappa\:\right)+\mathfrak{e}\left(\varkappa\:\right)\right]$$

Expanding the term $\:\mathfrak{e}\left(\varkappa\:+1\right)$ in a linear form using the Taylor series as

49 $$\:\mathfrak{e}\left(\varkappa\:+1\right)=\mathfrak{e}\left(\varkappa\:\right)+\frac{\partial\:\mathfrak{e}\left(\varkappa\:\right)}{\partial\:\mathcal{D}\left(\varkappa\:\right)}+HOT$$

Where $\:\mathcal{D}\left(\varkappa\:\right)\:$is the tunable parameter of the DRQNN model and HOT gives higher order terms that may be neglected. Thus Eq. (49) becomes

50 $$\:\mathfrak{e}\left(\varkappa\:+1\right)-\mathfrak{e}\left(\varkappa\:\right)=\varDelta\:\mathfrak{e}\left(\varkappa\:\right)=\frac{\partial\:\mathfrak{e}\left(\varkappa\:\right)}{\partial\:\mathcal{D}\left(\varkappa\:\right)}\varDelta\:\mathcal{D}\left(\varkappa\:\right)$$

Substituting into Eq. (48), then

51 $$\:\varDelta\:{\mathcal{V}}_{2}\left(\varkappa\:\right)=\left(\frac{\partial\:\mathfrak{e}\left(\varkappa\:\right)}{\partial\:\mathcal{D}\left(\varkappa\:\right)}\right)\varDelta\:\mathcal{D}\left(\varkappa\:\right)\left\{\frac{1}{2}\left[\frac{\partial\:\mathfrak{e}\left(\varkappa\:\right)}{\partial\:\mathcal{D}\left(\varkappa\:\right)}\varDelta\:\mathcal{D}\left(\varkappa\:\right)\right]+\mathfrak{e}\left(\varkappa\:\right)\right\}\:$$

For DRQNN, $\:\varDelta\:\mathcal{D}\left(\kappa\:\right)$ is given as

52 $$\:\varDelta\:\mathcal{D}\left(\varkappa\:\right)=\:{\lambda\:}_{\mathcal{i}\:}\mathfrak{e}\left(\varkappa\:\right)\frac{\partial\:\mathfrak{e}\left(\varkappa\:\right)}{\partial\:\mathcal{D}\left(\varkappa\:\right)}$$

where the modeling error $\:\mathfrak{e}\left(\varkappa\:\right)={\mathcal{y}}_{p}\left(\varkappa\:\right)-{\stackrel{\sim}{\mathcal{y}}}_{N}\left(\varkappa\:\right)$, So $\:\frac{\partial\:\mathfrak{e}\left(\varkappa\:\right)}{\partial\:\mathcal{D}\left(\varkappa\:\right)}=-\frac{\partial\:{\stackrel{\sim}{\mathcal{y}}}_{N}\left(\varkappa\:\right)}{\partial\:\mathcal{D}\left(\varkappa\:\right)}$. The term $\:\varDelta\:\mathcal{D}\left(\varkappa\:\right)$ in Eq. (52) is given by

53 $$\:\varDelta\:\mathcal{D}\left(\varkappa\:\right)=-\:{\lambda\:}_{\mathcal{i}\:}\mathfrak{e}\left(\varkappa\:\right)\frac{\partial\:{\stackrel{\sim}{\mathcal{y}}}_{N}\left(\varkappa\:\right)}{\partial\:\mathcal{D}\left(\varkappa\:\right)}$$

Using the above equation, the change in Lyapunov function can be obtained as

54 $$\:\varDelta\:{\mathcal{V}}_{2}\left(\varkappa\:\right)=-\left(\frac{\partial\:{\stackrel{\sim}{\mathcal{y}}}_{N}\left(\varkappa\:\right)}{\partial\:\mathcal{D}\left(\varkappa\:\right)}\right)\left(\:{\lambda\:}_{\mathcal{i}\:}\mathfrak{e}\left(\varkappa\:\right)\frac{\partial\:{\stackrel{\sim}{\mathcal{y}}}_{N}\left(\varkappa\:\right)}{\partial\:\mathcal{D}\left(\varkappa\:\right)}\right)\left\{\frac{1}{2}\left[\frac{\partial\:{\stackrel{\sim}{\mathcal{y}}}_{N}\left(\varkappa\:\right)}{\partial\:\mathcal{D}\left(\varkappa\:\right)}\left(-\:{\lambda\:}_{\mathcal{i}\:}\mathfrak{e}\left(\varkappa\:\right)\frac{\partial\:{\stackrel{\sim}{\mathcal{y}}}_{N}\left(\varkappa\:\right)}{\partial\:\mathcal{D}\left(\varkappa\:\right)}\right)\right]+\mathfrak{e}\left(\varkappa\:\right)\right\}\:$$

55 $$\:\varDelta\:{\mathcal{V}}_{2}\left(\varkappa\:\right)=\frac{1}{2}{\left[\frac{{\stackrel{\sim}{\mathcal{y}}}_{N}\left(\varkappa\:\right)}{\partial\:\mathcal{D}\left(\varkappa\:\right)}\right]}^{4}{\lambda\:}_{\mathcal{i}}^{2}{\mathfrak{e}}^{2}\left(\varkappa\:\right)-{\left[\frac{\partial\:{\stackrel{\sim}{\mathcal{y}}}_{N}\left(\varkappa\:\right)}{\partial\:\mathcal{D}\left(\varkappa\:\right)}\right]}^{2}{\lambda\:}_{\mathcal{i}\:}{\mathfrak{e}}^{2}\left(\varkappa\:\right)\:$$

56 $$\:\varDelta\:{\mathcal{V}}_{2}\left(\varkappa\:\right)=-{\lambda\:}_{\mathcal{i}\:}\left(\varkappa\:\right){\mathfrak{e}}^{2}\left(\varkappa\:\right){\left[\frac{\partial\:{\stackrel{\sim}{\mathcal{y}}}_{N}\left(\varkappa\:\right)}{\partial\:\mathcal{D}\left(\varkappa\:\right)}\right]}^{2}\:\left\{1-\frac{{\lambda\:}_{\mathcal{i}\:}\left(\varkappa\:\right)}{2}{\left[\frac{\partial\:{\stackrel{\sim}{\mathcal{y}}}_{N}\left(\varkappa\:\right)}{\partial\:\mathcal{D}\left(\varkappa\:\right)}\right]}^{2}\right\}\:$$

The condition of stability $\:\varDelta\:{\mathcal{V}}_{2}\left(\varkappa\:\right)\le\:0$, and it can be achieved as

57 $$\:{\lambda\:}_{\mathcal{i}\:}\left(\varkappa\:\right){\mathfrak{e}}^{2}\left(\varkappa\:\right){\left[\frac{\partial\:{\stackrel{\sim}{\mathcal{y}}}_{N}\left(\varkappa\:\right)}{\partial\:\mathcal{D}\left(\varkappa\:\right)}\right]}^{2}\:\left\{1-\frac{{\lambda\:}_{\mathcal{i}\:}\left(\varkappa\:\right)}{2}{\left[\frac{\partial\:{\stackrel{\sim}{\mathcal{y}}}_{N}\left(\varkappa\:\right)}{\partial\:\mathcal{D}\left(\varkappa\:\right)}\right]}^{2}\right\}\ge\:0$$

For the first term$\:\:{\lambda\:}_{\mathcal{i}\:}{\left(\varkappa\:\right)\mathfrak{e}}^{2}\left(\varkappa\:\right){\left[\frac{\partial\:{\stackrel{\sim}{\mathcal{y}}}_{N}\left(\varkappa\:\right)}{\partial\:\mathcal{D}\left(\varkappa\:\right)}\right]}^{2}$, the following condition of learning rate is obtained:

58 $$\:{\lambda\:}_{\mathcal{i}\:}\left(\varkappa\:\right)\ge\:0$$

And from the second term,$\:\left\{1-\frac{{\lambda\:}_{\mathcal{i}\:}\left(\varkappa\:\right)}{2}{\left[\frac{\partial\:{\stackrel{\sim}{\mathcal{y}}}_{N}\left(\varkappa\:\right)}{\partial\:\mathcal{D}\left(\varkappa\:\right)}\right]}^{2}\right\}$, $\:\varDelta\:{\mathcal{V}}_{2}\left(\varkappa\:\right)\le\:0$ if the following condition is satisfied

$$\:\left\{1-\frac{{\lambda\:}_{\mathcal{i}\:}\left(\varkappa\:\right)}{2}{\left[\frac{\partial\:{\stackrel{\sim}{\mathcal{y}}}_{N}\left(\varkappa\:\right)}{\partial\:\mathcal{D}\left(\varkappa\:\right)}\right]}^{2}\right\}\ge\:0\:\:\:\:\:\:\:\:\:\:\:\:\:or\:\:\:\:\:\:\:\:\left\{2-{\lambda\:}_{\mathcal{i}\:}\left(\varkappa\:\right){\left[\frac{\partial\:{\stackrel{\sim}{\mathcal{y}}}_{N}\left(\varkappa\:\right)}{\partial\:\mathcal{D}\left(\varkappa\:\right)}\right]}^{2}\right\}\ge\:0$$.

Thus we have

59 $$\:{\lambda\:}_{\mathcal{i}\:}\left(\varkappa\:\right)\le\:\frac{2}{{\left[\frac{\partial\:{\stackrel{\sim}{\mathcal{y}}}_{N}\left(\varkappa\:\right)}{\partial\:\mathcal{D}\left(\varkappa\:\right)}\right]}^{2}}$$

Finally, the stability is guaranteed if:

60 $$\:0\le\:{\lambda\:}_{\mathcal{i}\:}\left(\varkappa\:\right)\le\:\frac{2}{{\left[\frac{\partial\:{\stackrel{\sim}{\mathcal{y}}}_{N}\left(\varkappa\:\right)}{\partial\:\mathcal{D}\left(\varkappa\:\right)}\right]}^{2}}$$

Thus, choosing a learning rate within this range ensures that the Lyapunov function decreases, guaranteeing system stability.

## Simulation results

This section focuses on demonstrating the efficiency of the developed DRQNN-LS for nonlinear systems modeling. Three examples were employed here: nonlinear system identification, chaotic time series prediction, and the DC motor as a physical system application. The proposed method was validated through comparisons with DRNN-GD, DRNN-LS, and DRQNN-GD, enabling a clear assessment of the roles of recurrence, quantum modeling, and Lyapunov-based learning. The responses of the developed DRQNN-LS are compared to traditional methodologies such as DRNN-GD^44^, DRNN-LS^45^, and DRQNN-GD. All comparative algorithms were implemented in MATLAB under identical datasets, parameter settings, and simulation environments to ensure a fair performance evaluation. All the results are obtained using a laptop with a CPU of 12th Gen Intel (R) Core (TM) i7-1255U and 16 GB ram. To estimate the effectiveness of each model for comparison purposes, the root mean square error (RMSE), mean square error (MSE), and FIT indices defined as follows are used:

61 $$\:RMSE=\sqrt{\frac{1}{{\aleph\:}}{\sum\:}_{\varkappa\:=1}^{{\aleph\:}}{\left[{\mathcal{y}}_{p}\left(\varkappa\:\right)-{\stackrel{\sim}{\mathcal{y}}}_{N}\left(\varkappa\:\right)\right]}^{2}}$$

62 $$\:MSE=\frac{1}{{\aleph\:}}{\sum\:}_{\varkappa\:=1}^{{\aleph\:}}{\left[{\mathcal{y}}_{p}\left(\varkappa\:\right)-{\stackrel{\sim}{\mathcal{y}}}_{N}\left(\varkappa\:\right)\right]}^{2}$$

63 $$\:FIT\left(\mathrm{\%}\right)=\left(1-\frac{\parallel\widehat{\mathcal{y}}\left(\varkappa\:\right)-{\stackrel{\sim}{\mathcal{y}}}_{N}\left(\varkappa\:\right)\parallel}{\parallel\widehat{\mathcal{y}}\left(\varkappa\:\right)-\mathrm{m}\mathrm{e}\mathrm{a}\mathrm{n}\left(\widehat{\mathcal{y}}\right)\parallel}\right)\times\:100$$

These indices are characterized by the number of iterations for either the training or testing process expressed by $\:{\aleph\:}$. The RMSE is a useful statistic that assesses how well a model predicts values by allowing us to calculate the amount of error that exists between the anticipated and actual values. We can aim for more accurate and dependable forecasts by reducing RMSE. Moreover, larger errors are penalized more harshly by the MSE, which offers a thorough assessment of the model’s overall performance. Better model performance is shown by minimal MSE, which shows an improvement in the precision of the model’s outputs. Additionally, the FIT index is like a judge. It is used to compare our model’s output with real data to assess its performance. FIT index provides us a positive signal when our model accurately matches our data. If it doesn’t, they suggest a problem.

### Example 1

The dynamics of the nonlinear system employed here is characterized by the following difference equation^46^:

64 $$\:{\mathcal{y}}_{\mathrm{p}}\left(\varkappa\:+1\right)=0.72{\mathcal{y}}_{\mathrm{p}}\left(\varkappa\:\right)+0.025{\mathcal{y}}_{\mathrm{p}}\left(\varkappa\:-1\right)\mathcal{r}\left(\varkappa\:\right)+0.001{\mathcal{r}}^{2}\left(\varkappa\:-1\right)+0.2\mathcal{r}\left(\varkappa\:-2\right)$$

Where $\:{\mathcal{y}}_{\mathrm{p}}$ and $\:\mathcal{r}$ are the system’s output and input respectively. The identified DRQNN-LS model is represented in the following difference equation:

65 $$\:{\stackrel{\sim}{\mathcal{y}}}_{N}(\varkappa\:+1)=\widehat{\mathcal{f}}({\mathcal{y}}_{\mathrm{p}}\left(\varkappa\:\right),\:{\mathcal{y}}_{\mathrm{p}}\left(\varkappa\:-1\right),\:\mathcal{r}\left(\varkappa\:\right),\mathcal{r}\left(\varkappa\:-1\right),\mathcal{r}\left(\varkappa\:-2\right))$$

The prime objective to be achieved is estimating a nonlinear function $\:\widehat{\mathcal{f}}$ based on DRQNN-LS as the learning process progresses. Table 1 summarizes the hyperparameters for all models utilized here for Example 1.

**Table 1:** Model hyperparameters for example 1.

Model	Neurons per layer	Initialization Method	Learning rate ($\:{\lambda\:}_{\mathcal{i}\:})$ (Hidden, Output, Recurrent)
DRNN-GD^42^	(6,62,1)	initial random parameters	(0.0001, 0.0001, 0.0001)
DRNN-LS^43^	(6,61,1)	initial random parameters	(0.0006, 0.0006, 0.0006)
DRQNN-GD	(6,36,1)	initial random parameters	(0.77, 0.2, 1)
DRQNN-LS	(6,19,1)	initial random parameters	(0.04, 0.0508, 0.038)

#### Training phase

In the training phase, the following input is supplied to the given plant that is defined as

66 $$\:\mathcal{r}\left(\varkappa\:\right)=1.05\:\mathrm{s}\mathrm{i}\mathrm{n}\left(\frac{2{\uppi\:}\varkappa\:}{45}\right)\:\:\:\:\:\:\:\:\:\:\:\:\:0\le\:\varkappa\:\le\:1000$$

**Fig. 4:**
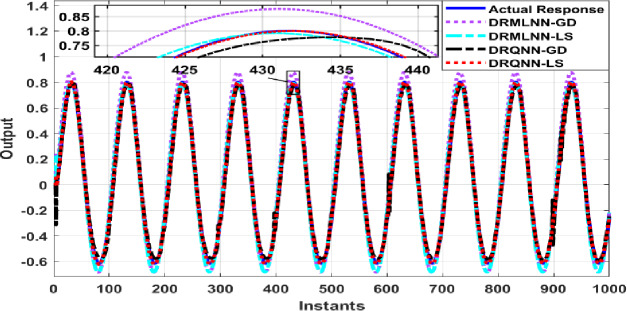
System response for the proposed DRQNN-LS during the training phase compared to other techniques [Example 1].

**Fig. 5:**
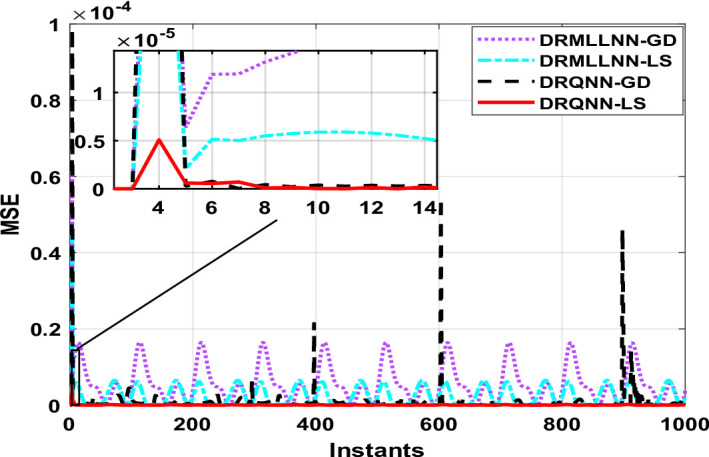
MSE during the training phase using DRQNN-LS model compared to other techniques [Example 1].

The generated information trains the developed DRQNN-LS modeling paradigm. Figure 4 shows the response of the early stage during the training process. It can be inferred that the response of the developed model based on LS tracks the actual response in a very early stage, which means that the proposed algorithm provides a very good and fast weight adjustment. Figure 5 depicts the equivalent MSE acquired in the same early the training phase. As seen here, the MSE gets reduced very fast and settles within a small range, getting a very small MSE. After small instants, it can be noticed that the response of DRQNN-LS being applied during this research fully tracks the actual output of the plant under study. In the training process, 1000 input/output data were generated to train the proposed model. The RMSE, MSE, FIT, and computation time are calculated as given in Table 2. The results of the developed DRQNN-LS are compared to those of DRQNN-GD, DRNN-LS, and DRNN-GD. It is obviously observed that the developed method here gives much lesser RMSE and MSE and the best FIT% value between all the methods, which indicates the superiority of the developed methodology in depicting dynamics of this nonlinear system accurately.

#### Testing phase

It should be noted that the DRQNN-LS operates in an online identification mode; hence, the adaptive weights continue to update during the testing phase according to the Lyapunov stability-based rule. This design enables the network to adapt to new inputs while maintaining stability. For testing the proposed algorithm for different input signals, the input signal defined in Eq. (67) is used in the testing stage. The test signal was chosen to check the effectiveness of DRQNN-LS under different amplitudes and frequencies, which is a good challenge.

67 $$\:\mathcal{r}\left(\varkappa\:\right)=\left\{\begin{array}{c}\:\:\:\:\:\:\:\:\:\:\:\:\:\:\:\:\:\:\:\:\:\:\:\:\:\:\:\:\:\:\:\:\:\:\begin{array}{cc}\mathrm{s}\mathrm{i}\mathrm{n}\left(\frac{2{\uppi\:}\varkappa\:}{50}\right)\:\:\:\:\:\:\:\:\:\:\:\:\:\:\:\:\:\:\:\:\:\:\:\:\:\:\:\:\:\:&\:\:\:\:\:\:0\:\le\:\varkappa\:<250\\\:1\:\:\:\:\:\:\:\:\:\:\:\:\:\:\:\:\:\:\:\:\:\:\:\:\:\:\:\:\:&\:250\:\le\:\varkappa\:<500\end{array}\\\:\begin{array}{cc}\:\:\:\:\:\:-1&\:500\:\le\:\:\varkappa\:<750\\\:0.3\:\mathrm{s}\mathrm{i}\mathrm{n}\left(\frac{2{\uppi\:}\varkappa\:}{50}\right)+0.1\:\mathrm{s}\mathrm{i}\mathrm{n}\left(\frac{2{\uppi\:}\varkappa\:}{46}\right)+0.6\:\mathrm{s}\mathrm{i}\mathrm{n}\left(\frac{2{\uppi\:}\varkappa\:}{20}\right)&\:750\le\:\varkappa\:\le\:1000\end{array}\end{array}\right.$$

**Fig. 6:**
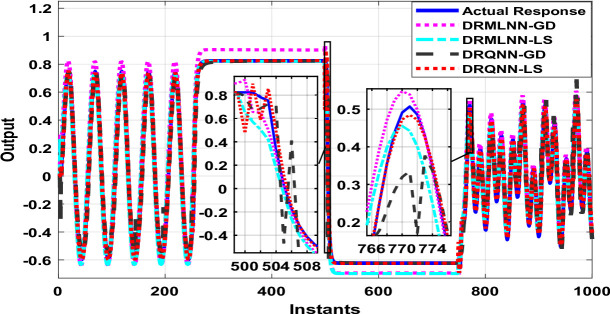
System response for the proposed DRQNN-LS during the testing phase compared to other techniques [**Example 1**].

**Fig. 7:**
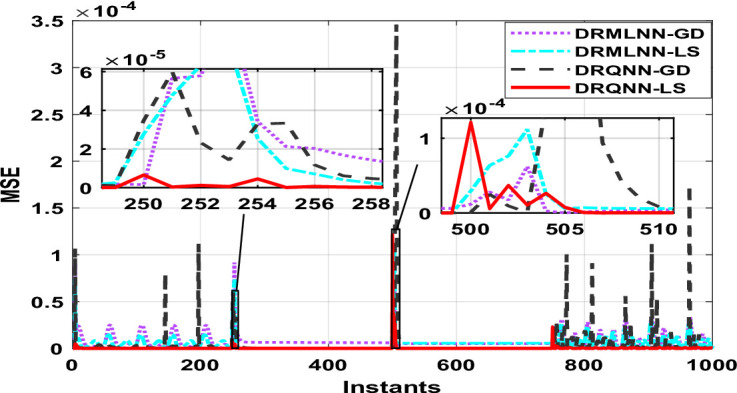
MSE during the testing phase using DRQNN-LS model compared to other algorithms [**Example 1**].

The output of DRQNN-LS during the testing phase is indicated in Fig. 6, and the corresponding MSE is depicted in Fig. 7. The MSE is reduced very fast even when the input sharply changes; the MSE gets reduced in a few instants, which indicates the ability of the learning algorithm to update the weights very fast. It is inferred that the output of the proposed algorithm tracks the actual response. The MSE shows the efficacy of the developed DRQNN-LS is assessed by comparing it with other algorithms during the testing phase in regard to RMSE, MSE, and FIT%, which is given in Table 2. It is inferred that the developed DRQNN-LS model gives the best modeling results (lowest RMSE and MSE and highest FIT%). The proposed DRQNN-based model exhibit slightly higher computational times during both training and testing compared to other architectures, which can be attributed to the inclusion of the quantum diagonal structure and the stable learning algorithm. The increase in computational time primarily stems from diagonal recurrent structure, while significantly reducing complexity compared to fully recurrent networks, still involves recurrent state updates and the online tuning of extra two parameters for quantum computation including the phase bias and reversal factor. These additional calculations, particularly the evaluation of Lyapunov functions and their derivatives, contribute to the observed increase in computation time.

**Table 2:** Comparative performance of DRQNN-LS and other models for example 1.

Model	DRNN-GD^44^	DRNN-LS^45^	DRQNN-GD	DRQNN-LS
Training RMSE	0.0790	0.0554	0.0302	0.0059
Training MSE	0.0062	0.0031	0.00091	3.44 × 10^− 5^
Training FIT%	85.1708	89.8974	95.2777	99.0011
Test RMSE	0.0828	0.0706	0.0590	0.0186
Test MSE	0.0069	0.0050	0.0035	3.45 × 10^− 4^
Test FIT%	85.9429	89.3138	94.3944	98.6331
Training computation time (s)	0.0053	0.0074	0.0164	0.0172
Test computation time (s)	0.0001	0.0009	0.0013	0.0029

The response of the DRQNN-LS is evaluated when the measured output contains white Gaussian noise included into the test input defined within Eq. (67). Three distinct standard deviation (STD) values for the noise (0.3, 0.5, 0.7) have been utilized with the test signal that affects the test response. The values of RMSE, MSE, and FIT% are calculated in Table 3 for the developed DRQNN-LS and other algorithms. The proposed DRQNN-LS is much better than other DRNN algorithms, even under different levels of noise.

The response results of this nonlinear system explain the ability of the developed paradigm DRQNN based on Lyapunov stability theory for modeling nonlinear systems. It is obvious that the developed methodology provides superior efficiency compared to all other algorithms. Also, the Lyapunov stability-dependent methods are not affected much by the noise added to the test output, unlike the gradient descent methods that give higher RMSE and MSE and lower FIT values for noisy data.

**Table 3:** Comparative performance of models with noisy data for example 1.

STD	Criterion	DRNN-GD^44^	DRNN-LS^45^	DRQNN-GD	DRQNN-LS
0.3	RMSE	0.1118	0.09	0.08	0.0598
	MSE	0.0125	0.0081	0.0064	0.0036
	FIT (%)	81.9142	86.2284	87.4340	90.7764
0.5	RMSE	0.1413	0.1248	0.0975	0.0928
	MSE	0.02	0.0156	0.0095	0.0086
	FIT (%)	77.3714	80.1683	84.6997	85.3634
0.7	RMSE	0.1749	0.1588	0.1309	0.1264
	MSE	0.0306	0.0252	0.0171	0.0160
	FIT (%)	72.0962	74.9117	79.2234	79.9842

### Example 2: chaotic Henon system modeling

In this application, the proposed method (DRQNN-LS) was used to infer the unpredicted output of the chaotic Henon plant. The discrete-time equation that describes the chaotic Henon system is given as^47^:

68 $$\:{\mathcal{y}}_{\mathrm{p}}\left(\varkappa\:+1\right)=-\mathcal{P}.{\mathcal{y}}_{\mathrm{p}}^{2}\left(\varkappa\:\right)+\mathrm{Q}.{\mathcal{y}}_{\mathrm{p}}\left(\varkappa\:-1\right)+1.0$$

The response of this system becomes a chaotic strange attractor when the parameters $\:\mathcal{P}=1.4$ and $\:\mathrm{Q}=0.3$ are utilized. To model this chaotic system, only the current system output $\:{\mathcal{y}}_{\mathrm{p}}\left(\varkappa\:\right)$ is used as the input to the DRQNN-LS algorithm developed here. The initial values of the system output were chosen to be $\:{\mathcal{y}}_{\mathrm{p}}\left(0\right)=0.4$ and $\:{\mathcal{y}}_{\mathrm{p}}\left(1\right)=0.4$ as described in^48^. The utilized hyperparameters of the models here for Example 2 are given in Table 4.

**Table 4:** Model hyperparameters for example 2.

Model	Neurons per layer	Initialization Method	Learning rate ($\:{\lambda\:}_{\mathcal{i}\:})$ (Hidden, Output, Recurrent)
DRNN-GD^42^	(1,107,1)	initial random parameters	(1.0000e-06, 1.0000e-06, 1.0000e-06)
DRNN-LS^43^	(1,108,1)	initial random parameters	(6.2600e-08, 6.2600e-08, 6.2600e-08)
DRQNN-GD	(1,49,1)	initial random parameters	(0.1, 0.06, 0.412)
DRQNN-LS	(1,84,1)	initial random parameters	(0.005, 0.01, 0.0491)

**Fig. 8:**
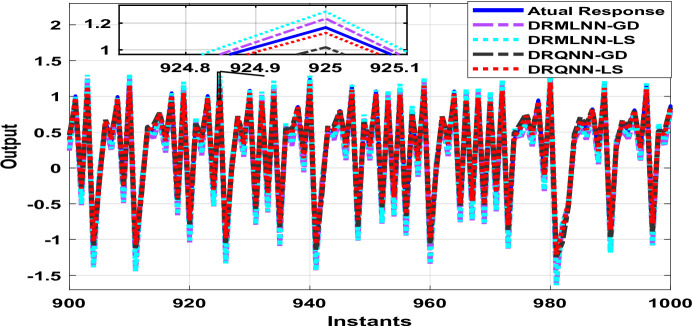
Response of the proposed DRQNN-LS during training compared to other techniques [**Example 2**].

**Fig. 9:**
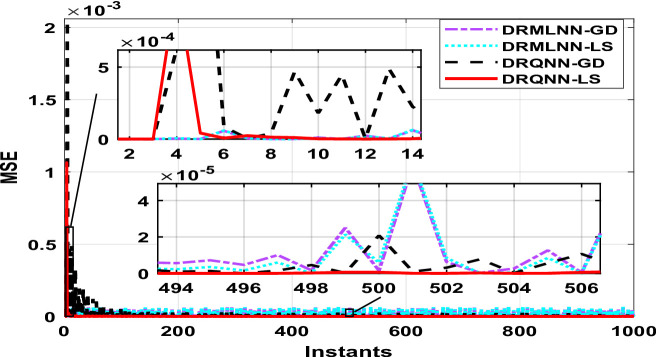
MSE with DRQNN-LS during the training phase compared to other algorithms [**Example 2**].

**Fig. 10:**
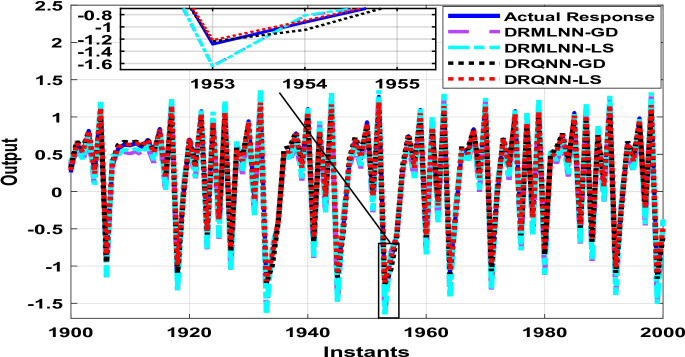
Response of DRQNN-LS during the testing phase compared to other paradigms [**Example 2**].

**Fig. 11:**
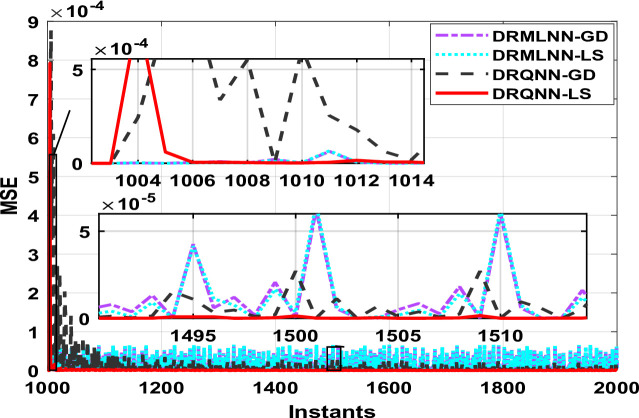
MSE with DRQNN-LS during the testing phase compared to other paradigms [**Example 2**].

Two thousand output data were generated, which are used to train and test the proposed algorithm. Testing is done on the remaining thousand samples, whereas training is done on the first thousand. During training, the response of the developed algorithm against actual output is demonstrated in Fig. 8. Here we only show the last 100 samples to show how well the DRQNN-LS output tracks the actual output. Figure 9 shows the MSE for all training data. Again, the Lyapunov stability method converges very fast to a very small error to force the model output to precisely follow the actual response of the plant. In the training phase, comparison among the suggested DRQNN-LS and other algorithms, including DRQNN-GD, DRMLNN-LS, and DRMLNN-GD, in regard to RMSE, MSE, FIT%, and computation times during training and testing are calculated as in Table 5. As seen in Table 5, the suggested algorithm achieves the highest efficacy compared to other algorithms. The other 1000 samples are used for testing the proposed algorithm, and its corresponding MSE is shown in Figs. 10 and 11. The corresponding performance indices for testing are given in Table 5.

For testing the proposed paradigm under different noise levels, the same three noise values used in the first example are employed here to show the robustness of DRQNN-LS for modelling the system even with noisy data. The same noise levels are also used for other DRNN algorithms utilized here for comparison. The values of RMSE, MSE, and FIT for the three noise levels are calculated in Table 6. The values depicted in Table 6 indicate how the proposed algorithm based on Lyapunov stability theory is robust to noise data and still gives the best performance of all other modelling methods.

Finally, the results obtained in both Tables 5 and 6 for this chaotic system show how well the proposed algorithm can be used to model even highly nonlinear systems even with noisy data. Therefore, the proposed DRQNN-LS can be used effectively to model highly nonlinear systems, especially chaotic systems, better than other models. The increased computational time for the proposed technique arises from the diagonal recurrent structure that involves recurrent states updates and the quantum computation including the tuning of two extra parameters based on Lyapunov stability criteria.

**Table 5:** Comparative performance of DRQNN-LS and other models for **Example 2.**

Model	DRNN-GD^44^	DRNN-LS^45^	DRQNN-GD	DRQNN-LS
Training RMSE	0.1166	0.1144	0.1125	0.0407
Training MSE	0.0136	0.0131	0.0127	0.0017
Training FIT%	89.1974	89.6826	91.8073	97.7958
Test RMSE	0.1184	0.1169	0.1092	0.0374
Test MSE	0.0140	0.0137	0.0119	0.0014
Test FIT%	89.039	89.352	91.6984	97.7654
Training comput-ation time (s)	0.0008	0.0011	0.0038	0.0093
Test computation time (s)	0.00046	0.00085	0.00092	0.0047

**Table 6:** Comparative performance of models with noisy data for **Example 2**.

STD	Criterion	DRNN-GD^44^	DRNN-LS^45^	DRQNN-GD	DRQNN-LS
0.3	RMSE	0.1381	0.1263	0.1258	0.0601
	MSE	0.0191	0.0160	0.0158	0.0036
	FIT (%)	87.9231	89.2203	91.9959	97.7827
0.5	RMSE	0.1605	0.1415	0.1449	0.0785
	MSE	0.0257	0.02	0.021	0.0062
	FIT (%)	87.1001	88.9964	91.7606	97.6527
0.7	RMSE	0.1858	0.1603	0.1661	0.0938
	MSE	0.0345	0.0257	0.0276	0.0088
	FIT (%)	86.5151	88.7972	91.6476	97.6495

### Example 3: modeling of DC motor

**Fig. 12:**
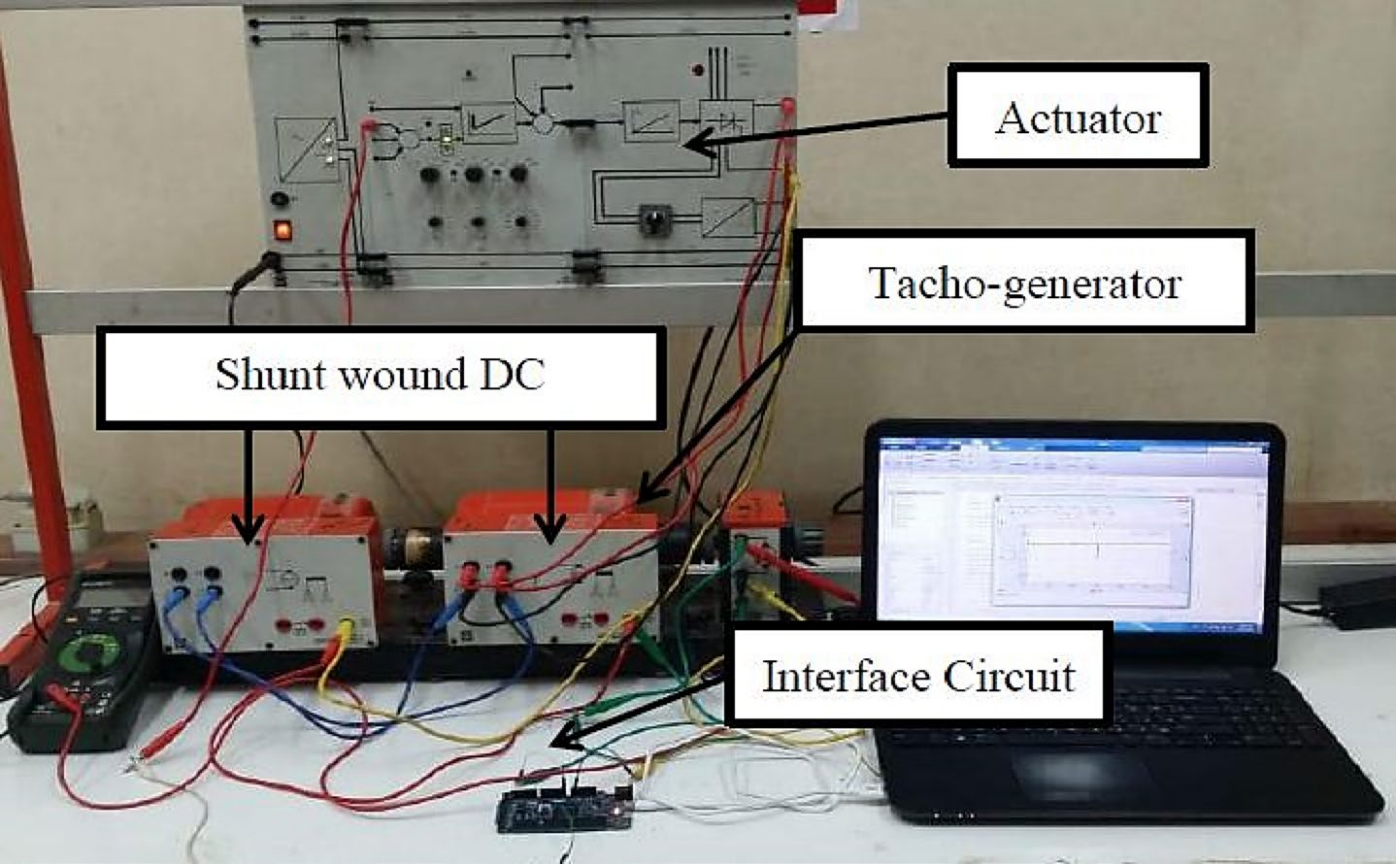
The plant’s experimental installation under study.

To demonstrate the capability of the suggested DRQNN to model a highly nonlinear practical plant even with noisy data. The proposed practical system here within this subsection is a shunt-wound DC motor of 0.1 kW. A mechanical clutch connects the two DC motors that make up the system being studied. One of them is to control the speed of the load, while the other acts as a nonlinear load applied to the shaft of the first motor. The plant’s experimental installation under study is shown in Fig. 12. The input is supplied to the motor, and its corresponding response is recorded through a tachogenerator whose transfer function is just a gain.

**Fig. 13:**
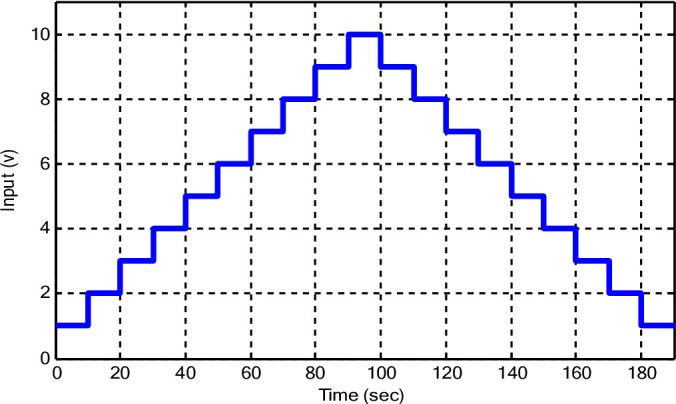
Input data for the training phase.

**Fig. 14:**
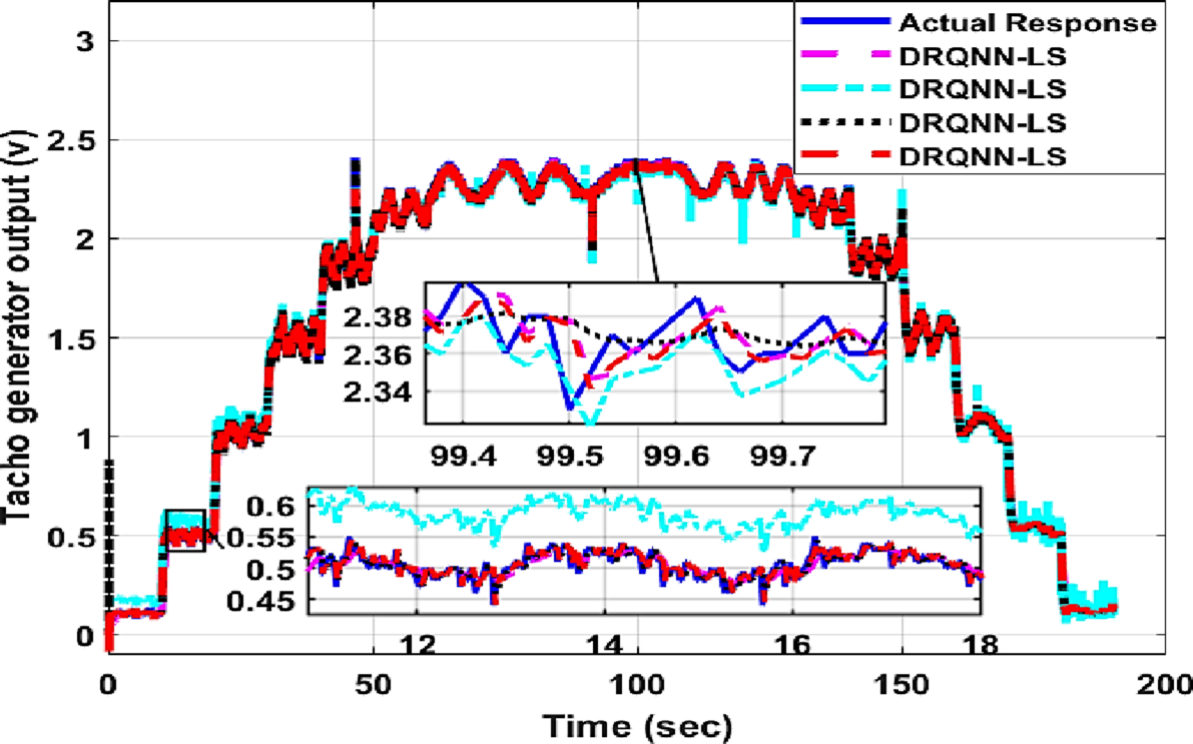
The output of the proposed DRQNN-LS during the training phase compared to other algorithms for the **DC motor**.

Input data is recorded for training the proposed algorithm as shown in Fig. 13. The corresponding output data versus the output of the suggested model is depicted in Fig. 14. It is obvious that the data recorded from the tachogenerator are noisy, which is normal for practical applications. The hyperparameters of the models employed here for DC are depicted in Table 7.

**Table 7:** Model hyperparameters for the DC motor.

Model	Neurons per layer	Initialization Method	Learning rate ($\:{\lambda\:}_{\mathcal{i}\:})$ (Hidden, Output, Recurrent)
DRNN-GD^42^	(4,2,1)	initial random parameters	(0.14, 0.14, 0.14)
DRNN-LS^43^	(4,13,1)	initial random parameters	(0.0108, 0.0108, 0.0108)
DRQNN-GD	(4,2,1)	initial random parameters	(0.003, 0.28, 1)
DRQNN-LS	(4,2,1)	initial random parameters	(0.01, 0.28, 1)

**Fig. 15:**
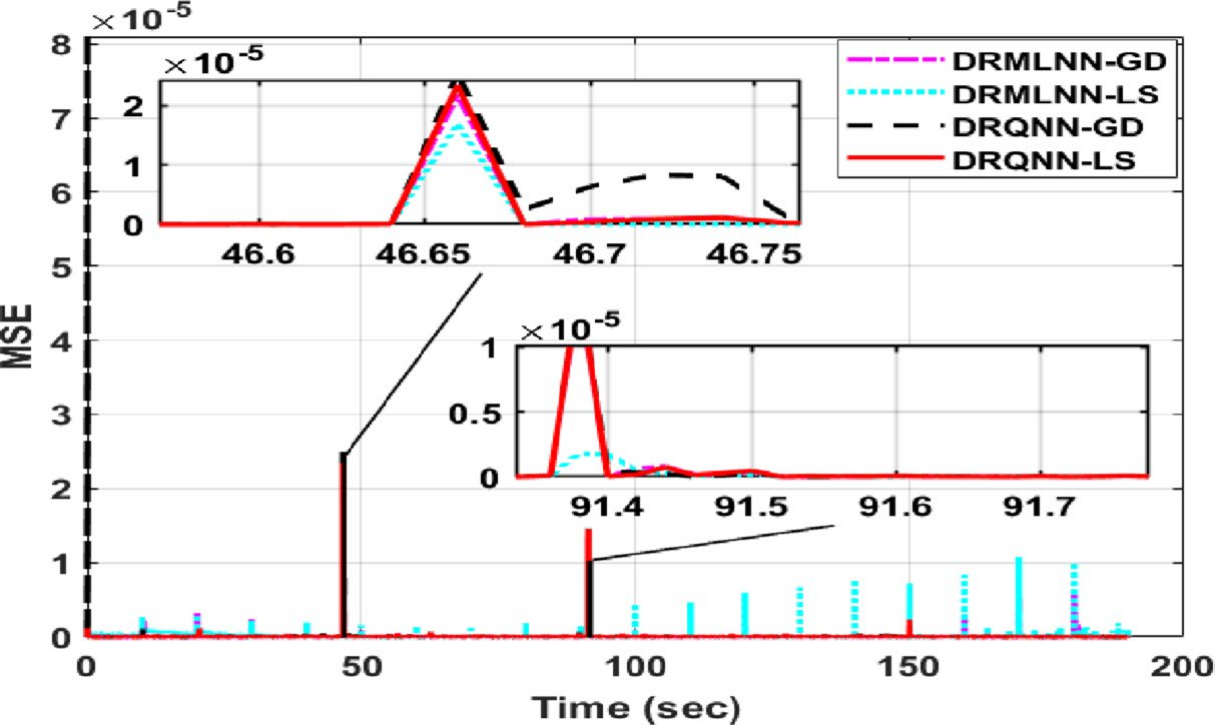
MSE obtained with the proposed method during the training phase compared to other techniques for the **DC motor.**

**Fig. 16:**
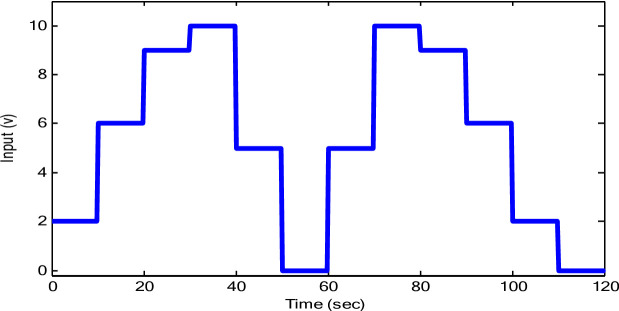
Input data for the testing phase.

**Fig. 17:**
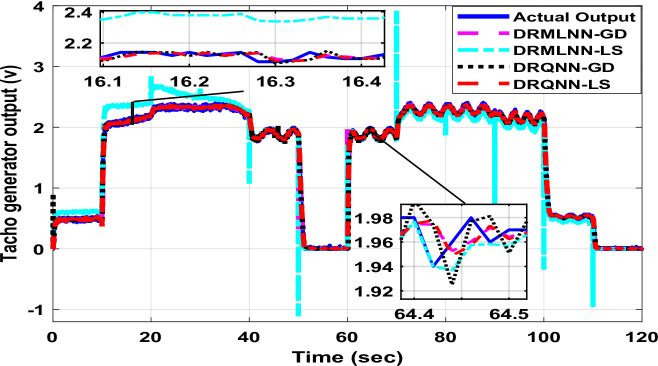
The output of the proposed DRQNN-LS during the testing phase compared to other techniques for the **DC motor.**

**Fig. 18:**
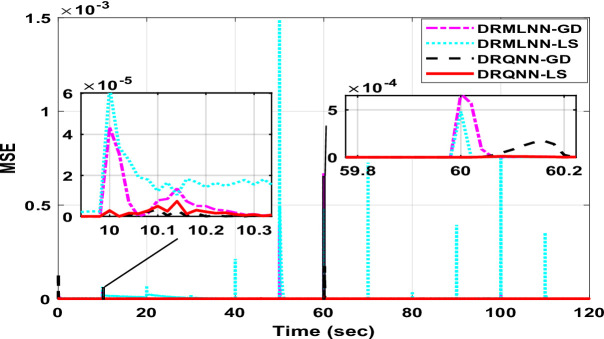
MSE obtained with the proposed method during the testing phase compared to other algorithms for the **DC motor.**

The response of the suggested DRQNN-LS tracks actual output precisely. The corresponding MSE is depicted in Fig. 15, which shows the efficacy of the suggested method for modeling nonlinear systems even with noisy data. Also, it depicts how fast the learning method based on Lyapunov theory updates the weights such that the DRQNN output tracks the actual response precisely. For the testing stage, the input voltage to the DC motor is depicted in Fig. 16. Figure 17 shows the DRQNN-LS model output versus the actual output from the tachogenerator. It is obvious that the response of the developed DRQNN-LS tracks the plant’s response precisely even under very sharp changes in the output. Figure 18 shows the MSE of the developed paradigm for the testing phase. Table 8 shows results of the suggested paradigm DRQNN-LS compared to DRQNN-GD, DRNN-LS, and DRNN-GD in terms of RMSE, MSE, FIT% and training and testing computation times. It is obvious that the efficacy of the suggested paradigm is the best compared to other approaches because it gives the least RMSE, MSE, and the best FIT% values. This illustrates the efficiency of the proposed method based on DRQNN-LS for identifying practical nonlinear systems compared to other RNN algorithms. However, the proposed DRQNN-LS impose additional calculations for updating the recurrent weights and extra parameters for quantum computation including phase bias and the reversal factor contribute to the observed increase in computation time.

**Table 8:** Comparative performance of DRQNN-LS and other models for the **DC motor.**

Model	DRNN-GD^44^	DRNN-LS^45^	DRQNN-GD	DRQNN-LS
Training RMSE	0.0214	0.0350	0.0212	0.0171
Training MSE	0.00046	0.0012	0.00045	0.000293
Training FIT%	97.9489	96.3694	98.0506	98.2135
Test RMSE	0.0541	0.0423	0.0360	0.0202
Test MSE	0.0029	0.0018	0.0013	0.000406
Test FIT%	98.1271	98.1321	98.1413	98.3444
Training computation time (s)	0.0007	0.0025	0.0031	0.0069
Test computation time (s)	0.00035	0.0013	0.0017	0.0023

Our simulation findings here in this part show that the suggested DRQNN-LS gives the best results in both the training and testing phases for all proposed systems here for free-noise and noisy data sets. Also, the identification of practical results of the DC motor shows how good and fast the proposed algorithm can identify the practical systems. This work focused on the identification of nonlinear dynamical systems, including but not limited to a time-varying system, a chaotic Henon series system, and a practical DC motor. For future work, the proposed algorithm will be utilized to control nonlinear systems and see its ability to reduce the effects of disturbances and parameter uncertainties. Overall, the results demonstrate that DRQNN-LS provides a well-balanced trade-off between computational efficiency and enhanced performance, confirming its suitability for dynamic system modeling.

The superior performance of the proposed DRQNN-LS over the compared methods stems from the combined effect of its diagonal recurrent quantum structure and Lyapunov-based learning mechanism. The diagonal recurrence reduces parameter coupling and internal instability while preserving the essential dynamic memory required for nonlinear system identification. In addition, the quantum-inspired neuron representation enhances the approximation capability for highly nonlinear dynamics compared to classical DRNN models. Most importantly, the Lyapunov-based adaptive learning rule guarantees stable weight updates and monotonic error reduction, avoiding the oscillations and slow convergence typically associated with gradient-descent-based methods. This synergy enables faster convergence, improved robustness to noise, and consistently lower RMSE/MSE and higher FIT values across all benchmark systems.

## Conclusion

This paper presented a novel Diagonal Recurrent Quantum Neural Network based on Lyapunov Stability (DRQNN-LS) for the identification of nonlinear dynamic systems. By integrating a diagonal recurrent architecture with quantum-inspired neuron representation and Lyapunov-based adaptive learning, the proposed framework achieves stable online learning, reduced parameter coupling, and enhanced nonlinear approximation capability. In this study, the convergence during the identification process has been satisfied by online updating the parameters using adaptive learning rates which have been derived based on Lyapunov stability. Tuning the extra two parameters related to the quantum computation increases the flexibility of the modeling technique design for handling the nonlinear dynamics offering superior performances. The effectiveness of the proposed DRQNN-LS was validated through comprehensive numerical simulations involving a nonlinear mathematical system, a chaotic Henon system, and a practical DC motor. Comparative analysis with DRNN-GD, DRNN-LS, and DRQNN-GD demonstrated that DRQNN-LS consistently outperforms existing approaches in terms of RMSE, MSE, and FIT indices, while maintaining robustness under noisy conditions. Although the proposed method introduces a modest increase in computational time due to recurrent state updates and Lyapunov-based learning, the reported execution times remain sufficiently small for real-time implementation using modern processors. Finally, DRQNN-LS scheme has potential capability to approximate highly nonlinear dynamics because the following reasons: (1) the developed structure has more degree of freedom, which depends on quantum computation and neural networks. (2) the developed learning algorithm in which the updating rules have been derived based on Lyapunov stability theorem. Despite the promising results, several directions remain open for future research. The current study is limited to single-input single-output (SISO) systems, and extending the DRQNN-LS framework to multi-input multi-output (MIMO) and large-scale nonlinear systems represents an important next step. Future work will also investigate the integration of the proposed identification framework into adaptive and robust control schemes. In addition, hardware-oriented implementations on real-time embedded platforms and emerging quantum or neuromorphic processors will be explored to further assess the practicality and scalability of the proposed approach.

## Data Availability

The datasets used and/or analysed during the current study available from the corresponding author on reasonable request.
